# Effects of breathing training on walking ability and quality of life in patients with multiple sclerosis: systematic review and meta-analysis of randomized controlled trials

**DOI:** 10.3389/fimmu.2025.1643938

**Published:** 2025-08-29

**Authors:** Cuiting Li, Jihe Kang, Xiuli Wang, Lulu Wang, Xiaoling Li

**Affiliations:** Department of Rehabilitation Medicine, Lanzhou University Second Hospital, Lanzhou, Gansu, China

**Keywords:** balance, breathing training, fatigue, multiple sclerosis, quality of life, sleep, walking ability

## Abstract

**Background:**

In recent years, an increasing number of studies have investigated the effects of breathing exercises on walking ability and quality of life in patients with multiple sclerosis (MS). However, the results of these studies are inconsistent. This study aims to explore the effects of breathing training on walking ability and quality of life in patients with MS through systematic review and Meta-analysis.

**Methods:**

PubMed, Web of Science, Embase and Cochrane English databases were systematically searched from inception to July 25 2025. Cochrane risk assessment tool and Physical Therapy Evidence database (PEDro) scale were used to evaluate the methodological quality of the included studies.

**Results:**

In total, 21 studies involving 881 patients were included. The 21 included studies demonstrated low risk of bias according to the Cochrane tool and high methodological quality (mean PEDro score = 6.24). The results revealed that, in terms of walking ability, breathing training significantly improved Berg Balance Scale (BBS) score (WMD, 3.25; 95%CI, 1.89 to 4.60, P < 0.00001, I^2^ = 58%). However, breathing training had no significant effect on the improvement of TUG (WMD, -0.96; 95% CI, - 2.12 to 0.20, P = 0.11, I^2^ = 0%) and 6WMT (WMD, 22.71; 95% CI, - 2.80 to 48.21, P = 0.08, I^2^ = 37%). In terms of quality of life, breathing training had a significant effect on measuring fatigue in patients with MS using FSS and MFIS assessments (SMD, -0.05; 95% CI, -0.76 to-0.24, P =0.0001, I^2^ = 57%)), but did not significantly improve the quality of life measured by SF-36 and MSQOL-54 (SMD, 0.15; 95%CI, -0.08 to 0.38, P= 0.19, I^2^ = 26%). In addition, breathing training had no significant effect on PSQI (WMD, -0.68; 95%CI, − 2.04 to 0.68, P= 0.33, I^2^ = 0%).

**Conclusions:**

Breathing training improves the balance function and alleviates fatigue symptoms in patients with MS, but there is no evidence to support its effects on functional mobility, overall quality of life and sleep quality. In the future, standardized breathing training programs should be established, and their long-term benefits for comprehensive functional rehabilitation of patients with MS should be further verified.

**Systematic review registration:**

https://www.crd.york.ac.uk/PROSPERO/, identifier CRD42024600560.

## 1 Introduction

Multiple sclerosis (MS) is an acquired inflammatory and disabling disease of the central nervous system (CNS) (1). In CNS regions affected by inflammation, neural signal transmission is disrupted (due to demyelination/axonal damage) (2). As a result, patients have sensory disorders, motor dysfunction, sleep, fatigue, cognitive and psychological symptoms, and also significantly affect breathing function, which in turn reduces walking ability and quality of life (3–7). 64% of people with MS and a low to medium Expanded Disability Status Scale (EDSS) score (3.96 ± 1.8, range 2.0 to 6.5; score range 0‐10) had breathing muscle weakness (8). The weakness of the breathing muscles related to MS is essentially a pathological cascade reaction resulting from both nerve damage and insufficient physical activity, along with complications of the breathing system (9). The progressive decline in motor ability and persistent fatigue throughout the course of the disease can further exacerbate the limitation of physical activity and the impairment of lung function (10). Therefore, specialized training for the breathing muscles may have potential clinical significance.

Clinically, by using pharmacological treatments that modulate the immune system and reduce inflammation, to delay the progression of the disease and reduce the disability rate (11). But at the same time, patients have a series of drug reactions, such as pain, allergy, mood swings, gastrointestinal reactions, etc. (12). Breathing problems are also often overlooked because they are not prominent in patients with MS in the early stage (13). However, with the progression of the disease, some patients will develop breathing dysfunction due to breathing muscle weakness, which is positively correlated with the degree of disability (14). It further aggravates the decline of exercise volume and fatigue of patients, increases the risk of falls, and has a huge impact on the balance, walking and daily life of patients (15). In recent years, rehabilitation intervention, especially breathing training, has played an increasingly significant role in the multi-dimensional and personalized comprehensive management of patients with MS (16). At present, studies have shown the effects of breathing muscle training on pulmonary function in patients with MS (17), and also explored the effects of exercise training on walking ability and quality of life in patients with MS (18). It is worth noting that current studies have shown that breathing training can improve the quality of life of stroke patients, which lays a foundation for the study of breathing training on the quality of life of patients with MS (19).

Researchers have explored the effects of breathing training on walking ability and quality of life in patients with MS, but the results of existing studies are conflicting. Ghannadi et al. (20) showed that breathing training not only increased lung function, but also significantly improved fatigue and all quality-of-life related indicators, but there was no significant change in the results of the 6-minute walking test. Khadadah et al. (21) have shown that breathing training can reduce fatigue and sleepiness associated with quality of life at 3 months, however, there were no statistically significant changes in fatigue, sleep quality, or quality of life after 6 months of treatment. Kezele et al. (22) have shown that breathing training can reduce fatigue, insomnia and mental illness. This study conducted a comprehensive systematic review and meta-analysis of randomized controlled trials to investigate the effects of respiratory training on the balance, walking ability, sleep, fatigue, and quality of life of patients with MS. From another perspective, the results of this study will have direct clinical application value. If this study can prove that breathing training has a significant effect on improving walking ability and quality of life in patients with MS, breathing function assessment can be incorporated into MS routine rehabilitation assessment system, at the same time, standardized breathing training for patients with MS was carried out.

## 2 Methods

This systematic review and meta-analysis was done in accordance with the Preferred Reporting Items for Systematic Reviews and Meta-Analysis (PRISMA, 2020) guideline (23). The protocol was registered with PROSPERO (CRD42024600560).

### 2.1 Search strategy

Search for randomized controlled trials (RCTs) in PubMed, Web of Science, Embase and Cochrane databases regarding the effects of breathing training on balance, walking, sleep, fatigue and quality of life in patients with MS. The search time was from inception to July 25, 2025. We also manually searched the literature from clinical registries. There are no publication dates, ages or set limits, but only articles published in English are included. Potentially relevant literature was searched in each database using subject headings combined with free words, and keywords were constructed according to study population, intervention method, and randomized controlled trials, such as: “multiple sclerosis”, “breathing training”, and “randomized controlled trials”. The complete search strategy is presented in **Supplementary Material**. This study complied with all PRISMA guidelines and the required information is reported accordingly. According to predetermined inclusion and exclusion criteria, two professional researchers in rehabilitation medicine independently screened each item for relevance based on title and abstract. Finally, the results of two authors were compared, and if the screening results were different, the third author would review. Final inclusion was determined by consultation among the three authors.

### 2.2 Inclusion and exclusion criteria

Inclusion criteria: (1) Study type: randomized controlled trial (RCT) on the effects of breathing training on patients with MS; (2) Subjects: patients who met the international diagnostic criteria for MS (24), were clearly diagnosed as MS, with stable condition, and had no age or gender restrictions; (3) Intervention measures: the experimental group only received breathing training or combined it with other basic treatment methods (such as regular training, standard care, medication, etc.), while the control group received sham breathing training, regular training, standard care, or medication, etc. (requiring balance of medications between groups); (4) Outcome indicators: (1) The main indicators of walking ability include the 6-minute walk test (6WMT), berg balance scale (BBS); the secondary indicators include the timed up and go test (TUG). (2) The main outcome indicators of quality of life: short Form 36 health survey (SF-36), MS quality of life - 54 questionnaire (MSQOL-54), fatigue severity sale (FSS), modified fatigue impact scale (MFIS); the secondary indicators are the Pittsburgh sleep quality index (PSQI). In order to investigate the effects of breathing training on walking ability and quality of life in patients with MS, we included yoga, Pilates, aerobic training, and power cycling training. Exclusion criteria: (1) duplicate publications; (2) non-randomized controlled trials such as self-control, retrospective studies, cohort studies and animal experiments; (3) The study participants were people without MS, and the intervention measures were non-breathing exercise. (4) the full text cannot be obtained by various means, the data in the study cannot be used, or the data cannot be converted into the form of mean ± standard deviation (Mean ± SD) by formula; (5) outcome indicators were unknown or lack of corresponding outcome indicators, baseline data were not comparable or baseline data were not reported; (6) non-English literature, (7) studies that adjusted the drug treatment plan during the research period were excluded.

### 2.3 Trial selection and data extraction

The retrieved literature was imported into Endnote, and two researchers independently screened the obtained literature according to the title of the abstract and the type of research according to the above inclusion and exclusion criteria. After excluding obviously irrelevant literature, the literature that met the inclusion criteria was screened and the full text was downloaded. Read the full text carefully and strictly follow the inclusion and exclusion criteria to complete the re-screening. Finally, data were extracted and checked from the included literature, and differences in results were resolved by discussion or consultation with a third researcher. The extracted content included: general information of the included literature (first author, publication year) and included studies (age, gender, duration of disease, number of cases, EDSS), treatment-related information (intervention, intervention time), Outcome indicators (including baseline and post-intervention measurements, reported as Mean± SD) were categorized into two dimensions: (1) Functional mobility: walking ability (6MWT); balance function (BBS and TUG). (2) Quality of life and related symptoms: quality of life (SF-36 and MSQoL-54); fatigue level (MFIS and FSS); sleep quality (PSQI).

### 2.4 Literature quality assessment

The quality of the included studies was assessed independently by two reviewers using the Cochrane risk of bias tool for Randomized Controlled Trials (25) and Physiotherapy Evidence Database (PEDro) scale (26). In case of disagreement between the two investigators, a third investigator joined the discussion until a consensus was reached among the three. Bias assessment included: (1) selection bias: random sequence generation; (2) selection bias: allocation concealment; (3) implementation bias: blinding of subjects and participants; (4) measurement bias: outcome assessors were blinded; (5) attrition bias: incomplete outcome data; (6) re-porting bias: selective reporting of results; (7) other biases. Each risk of bias was divided into low risk of bias, high risk of bias and unknown risk. PEDro is a core tool for assessing the methodological quality of randomized controlled trials (RCTS) in the field of physical therapy (27). It contains 11 assessment criteria, of which the first one is the screening criterion and the last 10 are the scoring items. 9–10 points: very high methodological quality (strictly meeting the difficult criteria of blinding and allocation concealing), 6–8 points: High quality (possible partial blinding or missing follow-up), score 4-5: moderate quality (significant methodological flaws), score 0-3: low quality (missing randomization or key design) (28).

### 2.5 Statistical analysis

In this study, a meta-analysis was performed using RevMan 5.3 software. All included indicators were uniformly converted into continuous variables, presented as Mean ± SD. For continuous outcomes, data obtained with the same measurement tool were displayed using mean difference (MD); data from different measurement methods were presented using standardized mean difference (SMD) along with its 95% confidence interval (95% CI). All outcome indicators reported in two or more studies were included in the analysis, specifically 6WMT, BBS, TUG, SF-36, MSQOL-54, FSS, MFIS, and PSQI. For each study, the means and standard deviations at baseline and after intervention were extracted to calculate the mean difference (MD) and its 95% confidence interval (CI). If data were reported as median and interquartile range (IQR), a specific conversion method was applied: the median was regarded as the mean, and the standard deviation was calculated as IQR/1.35 (29). The heterogeneity of the included studies was expressed by I^2^ value and P value. If I^2^ < 50% and P > 0.1, there was no significant heterogeneity among the studies, and the fixed effect model was used for Meta-analysis. If I^2^≥50% and P ≤ 0.1, it represented that there was heterogeneity between the study groups, and the heterogeneity was within the acceptable range, and the random effect model was used for analysis (30). In the subgroup analysis, the effects of age (young group, < 45 years old; middle-aged and elderly group, ≥ 45 years old), fatigue detection type (FSS and MFIS), and intervention type (aerobic training, Pilates, traditional breathing training) on fatigue were analyzed. At the same time, the effects of age (young group, < 45 years old; middle-aged and elderly groups, ≥ 45 years old), quality of life type (SF-36 and MSQol-54), and intervention type (aerobic training and traditional breathing training) on quality of life were also analyzed. RevMan 5.3 software was used to generate the forest plot. Statistical significance was considered for outcomes with a P<0.05 (31).

## 3 Results

### 3.1 Study selection

**Figure 1:**
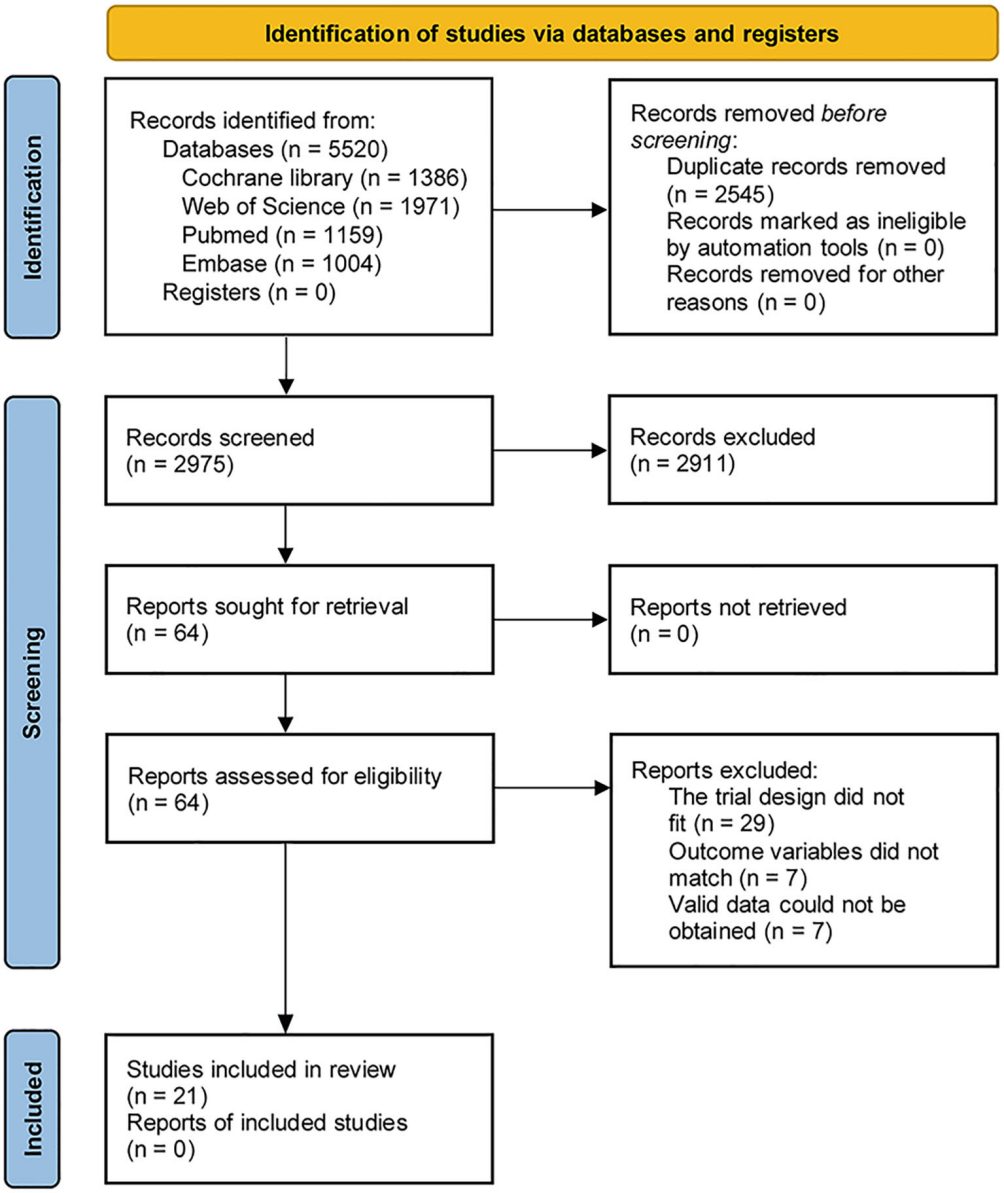
PRISMA flow diagram of the study selection process.

The literature screening process is shown in **Figure 1**. A total of 5520 articles were retrieved, including 1159 articles from PubMed, 1004 articles from Embase, 1971 articles from Web of Science and 1386 articles from Cochrane. No clinical registration center was searched manually. A total of 2545 duplicate articles were excluded, and then 2911 articles were excluded after reading the title and abstract. According to the inclusion and exclusion criteria, 64 studies were excluded after reading the full text: (1) the trial design did not fit (n=29); (2) outcome variables did not match (n=7); (3) Valid data could not be obtained (n=7), 21 articles (15, 20–22, 32–48) were finally included for systematic review and meta-analysis.

### 3.2 Characteristics of the included studies

Characteristics of the participants and interventions are shown in **Table 1**. A total of 21 studies were included, including 27 breathing training groups with 482 patients with MS and 21 control groups with 375 patients with MS. One study involved women (48), and 20 studies involved both men and women (15, 20–22, 32–47). The average age was 31.9 to 53.9 years. The intervention consisted of yoga intervention 5 times (32, 34, 35, 38, 48), aerobic and breathing muscle training intervention 2 times (22, 38), aerobic training intervention 9 times (32, 35–37, 41, 42, 44, 47, 48), Pilates intervention 5 times (15, 39, 43, 45, 46), breathing muscle training intervention 1 times (20), Baduanjin intervention 1 time (34), inspiratory muscle threshold training device 2 times (33, 40), and continuous positive airway pressure 1 time (21). Data on walking ability were provided in 11 (15, 20, 32–34, 38, 39, 44–46, 48)of the 21 studies (15, 20–22, 32–48), with 5 in BBS (34, 38, 39, 45, 48), 5 in TUG (20, 32, 39, 44, 46), and 8 in 6WMT (15, 20, 32, 33, 38, 44–46). In addition, there were 17 studies (20–22, 33, 35–45, 47, 48) that provided data related to QOL, including 3 for PSQI, 7 for FSS (21, 33, 36, 40, 41, 45, 48), 9 for MFIS (20, 22, 37–39, 41–44), 4 for SF-36 (20, 35, 38, 42), and 3 for MSQol-54 (21, 44, 45).

**Table 1:** Characteristics of the studies included in this meta-analysis.

Author & year	Region	Sample size	Sex (w/m)	Age (y)	EDSS	Disease duration (y)	Intervention	Details of interventions	Outcome measures	Primary outcome in original study
Farajnia et al.2025 (44)	Iran	IG=10	7/3	34.30 ± 8.42	1.45 ± 0.59	7.80 ± 5.11	Aerobic training	Ten weeks,20 sessions, 35–55 minutes/session	TUG,6WMT,MFIS,MSQOL-54	Brythonic training reduces IL-17 and outperforms control in TUG, 6MWT, fatigue, MSQOL-5.
		CG=10	8/2	41.30 ± 9.98	1.55 ± 0.69	9.90 ± 6.31	control group	daily routine		
Eldemir et al., 2024 (45)	Turkey	IG=15	14/1	41 ± 7.82	1.5 ± 1.23	10 ± 5.73	Pilates-TR	6 weeks, 3 days/week	BBS, 6MWT, FSS, MSQOL-54	Pilates can significantly improve muscle strength, core function, BBS, 6MWT, FSS and MSQOL-54 in patients with MS.
		CG=15	14/1	38.± 10.86	1.5 ± 1.64	8 ± 5.73	Usual care	normal lifestyle		
Lysogorskaia et al., 2023a (38)	Russia	IG=15	10/5	39 ± 10.4	4 ± 1.15	12.6 ± 8.4	yoga	12 weeks, 60–75 min. twice a week	BBS, 6WMT, SF-36, MFIS	Yoga, PT, and control group showed no significant differences in BBS, 6WMT, MFIS. However yoga performed significantly better than the other two groups in terms of SF-36.
		CG=12	11/1	46.2± 10.4	4.5 ± 1.07	18.5 ± 7.9	Usual care	normal lifestyle		
Lysogorskaia et al., 2023b (38)	Russia	IG=9	9/0	46.1± 11.3	4 ± 1.51	18.1 ± 12.3	aerobic and breathing training	12 weeks, 60–75 minutes, twice a week	BBS, 6WMT, SF-36, MFIS	
		CG=12	11/1	46.2± 10.4	4.5 ± 1.07	18.5 ± 7.9	Usual care	normal lifestyle		
Kezele et al., 2023 (22)	Croatia	IG=13	8/5	50.0 ± 9.3	3.8± 1.8	NR	aerobic and breathing training	8 weeks, 60 min/session, 2d/week	PSQI, MFIS	Compared with the control group, the training group showed significant reductions in ISI and CORE-OM, and significant improvements in PSQI and MFIS scores.
		CG=11	6/5	53.8 ± 11.8	4.0 ± 2.0	NR	Usual care	Normal lifestyle and visit the MSSC 8 weeks, 2d/week		
Pan et al., 2022a (34)	China	IG=30	22/8	42.23± 5.14	2.95 ± 0.74	6.23 ± 2.25	Baduanjin	24 weeks, 60minutes, once a day	BBS	Baduanjin and yoga both improve BBS, TIS, and FSS in MS patients; Baduanjin is more effective in enhancing dynamic balance and relieving depression.
		CG=20	14/6	42.25 ± 4.52	2.90 ± 0.77	5.44 ± 2.78	Usual care	normal lifestyle		
Pan et al., 2022b (34)	China	IG=30	21/9	40.93± 4.76	2.80 ± 0.87	5.15 ± 1.95	yoga	24 weeks, 60 minutes, once a day	BBS	Baduanjin and yoga both improve BBS, TIS, and FSS in MS patients; Baduanjin is more effective in enhancing dynamic balance and relieving depression.
		CG=20	14/6	42.25 ± 4.52	2.90 ± 0.77	5.44 ± 2.78	Usual care	normal lifestyle		
Khadadah et al., 2022a (21)	Canada	IG=17	11/6	49.6 ± 10	4.2 ± 1.8	21.2 ± 10.1	continuous positive airway pressure	3 months	FSS, PSQI, MSQOL-54	CPAP treatment did not significantly improve FSS in multiple sclerosis, but it could reduce ESS at 3 months and morning fatigue at 6 months, with no significant impact on MSQOL-54.
		CG=17	11/6	52.8 ± 8.8	3.9 ± 1.5	19.8 ± 11	placebo	3 months		
Ghannadi et al., 2022 (20)	Iran	IG=17	13/4	36.47 ± 7.62	3.52 ± 0.94	NR	respiratory muscle training	8 weeks, twice a day for three sets,15 repetitions/set	6WMT, TUG, MFIS, SF36	Respiratory muscle training significantly enhances respiratory muscle strength, improves pulmonary function, reduces MFIS and increases SF-36, with no significant differences in TUG and 6WMT.
		CG=19	14/5	39.36 ± 9.83	3.07 ± 0.59	NR	Usual care	normal lifestyle		
Fleming et al., 2021 (43)	Ireland	IG=29	27/2	45.3 ± 8.6	NR	NR	home-based Pilates (DVD)	8 weeks, 48 hours apart, twice a week,	MFIS	Home-based Pilates training can significantly improve anxiety, depressive symptoms, and MFIS in patients with multiple sclerosis.
		CG=34	27/7	48.2 ± 9.76	NR	NR	Usual care	normal lifestyle		
Abasıyanık et al., 2020 (15)	Turkey	IG=16	12/4	42.50 ± 6.76	3.06 ± 1.65	12.59 ± 6.23	Pilates and home training	8 weeks, once a week, plus two days of home training	6WMT	Pilates significantly outperforms home exercise in improving 6MWT, core stability, respiratory muscle strength, and cognitive function in patients with MS.
		CG=17	11/6	48.24 ± 11.79	3.24 ± 1.77	9.83 ± 8.7	Telephone follow-up	once a week		
Young et al., 2019a (32)	American	IG=27	22/5	49.67 ± 9.40	NR	13.56 ± 8.26	aerobic exercise	12 weeks,60 minutes, 3 times/week	TUG,6WMT	M2M significantly improved TUG and 6MWT in MS patients; AY had no significant effect. Neither significantly improved PROMIS fatigue or pain interference.
		CG=28	24/4	47.2 ± 10.33	NR	13.38 ± 8.50	life education information newsletter	Biweekly		
Kezele et al., 2019 (42)	Croatia	IG=10	4/6	53.9± 10.7	5.5 ± 1.75	NR	aerobic exercise	4weeks, 60 min/session,2 days/week,	MFIS, SF-36	Combined upper and lower limb with breathing training can significantly improve MFIS (fatigue) and SF-36 (quality of life) related indicators in patients with multiple sclerosis.
		CG=9	3/6	48.2 ± 9.3	5.6 ± 1.63	NR	Usual care and Visit the MSS	2 days/week		
Al-Sharman et al., 2019 (47)	Jordan	IG=17	13/4	38.7 ± 13	2.1 ± 1.8	9.6 ± 8.49	aerobic exercise	6 weeks, 50–60 minutes, three times a week	PSQI	Aerobic exercise can significantly improve the PSQI and objective sleep quality in patients with MS.
		CG=13	10/3	31.9 ± 10	1.9 ± 1.03	5.43 ± 4.2	nonaerobic exercises	6 weeks, 50–60 minutes, three times a week		
Duff et al., 2018 (46)	American	IG=15	12/3	45.7 ± 9.4	NR	NR	Pilates and massage therapy	12 weeks, 50 minutes, 2 times/week, once a week	6WMT, TUG	Pilates training can significantly improve walking ability (6MWT) and functional mobility (TUG) in patients with multiple sclerosis.
		CG=15	11/4	45.1 ± 7.4	NR	NR	massage therapy	Once a week		
Heine et al., 2017 (41)	Netherlands	IG=43	32/11	43.1 ± 9.8	2.5± 0.38	7.0 ± 2	aerobic interval training	16 weeks,30 minutes, three times/week	FSS, MFIS	Aerobic training can slightly improve MFIS and FSS in patients with multiple sclerosis and severe fatigue, but has no significant impact on social participation.
		CG=46	33/13	48.2 ± 9.2	3.0 ± 0.5	12 ± 4.25	Usual care	normal lifestyle		
KüçüK et al., 2016 (39)	Turkey	IG=11	7/4	47.2 ± 9.5	3.2± 2.2	14.8 ± 7.4	Pilates	8 weeks, 45–60 minutes,2 times/week	BBS, TUG, MFIS	Clinical Pilates better improved PASAT and quality of life in MS patients compared to the control group, with significant improvements in BBS, MFIS and MSFC.
		CG=9	6/3	49.7 ± 8.9	2.8± 1.4	14.2 ± 9.5	Usual care	normal lifestyle		
Ahmadi et al., 2013a (48)	Iran	IG=10	10/0	36.80± 9.17	2.40 ± 1.24	5.60 ± 3.30	aerobic training	8 weeks,50minutes, 3times/week	BBS, FSS	Both aerobic training and yoga can improve BBS, FSS, and walking endurance in patients with multiple sclerosis. Aerobic training is more beneficial for improving walking speed, while yoga is superior in relieving anxiety.
		CG=10	11/0	36.70± 9.32	2.25± 1.25	5.00 ± 3.05	Usual care	normal lifestyle		
Ahmadi et al., 2013b (48)	Iran	IG=11	11/0	32.27± 8.68	2 ± 1.09	4.72 ± 5.62	yoga	8 weeks,60–70 minutes, 3times/week	BBS, FSS	
		CG=10	11/0	36.70± 9.32	2.25± 1.25	5.00 ± 3.05	Usual care	normal lifestyle		
Pfalzer et al., 2011 (33)	USA	IG=20	18/2	49.6 ± 9.5	4.1 ± 1.9	NR	Threshold inspiratory muscle trainer	10 weeks, 15 minutes/day, three groups,15 replicates/group	FSS,6WMT	Inspiratory muscle training can significantly enhance inspiratory muscle strength and balance ability in patients with multiple sclerosis, with a tendency to improve 6MWT.
		CG=19	13/6	46.0 ± 9.8	3.2 ± 1.2	NR	Usual care	normal lifestyle		
McCullagh et al., 2008 (37)	Ireland	IG=17	14/3	40.5 ± 12.68	5.4 ± 4.35	NR	aerobic exercise	12weeks, 50minutes,2 times/week	MFIS	At 3 months, the exercise group showed greater improvements in HR, QOL, and MFIS. At 6 months, only the differences in FAMS and MFIS change scores remained significant.
		CG=13	10/3	33.58 ± 6.1	5 ± 3.52	NR	Usual care	normal lifestyle		
Oken et al., 2004a (35)	American	IG=22	20/2	49.8 ± 7.4	3.2 ± 1.7	NR	Yoga	26weeks,90 minutes, once a week	SF-36	The yoga and exercise groups showed significant improvements in SF-36 and MFIS, with no significant changes in mood indicators.
		CG=20	20/0	48.4 ± 9.8	3.1± 2.1	NR	Usual care	normal lifestyle		
Oken et al., 2004b (35)	American	IG=15	13/2	48.8± 10.4	2.9± 1.7	NR	aerobic exercise	one class per week along with home exercise	SF-36	The yoga and exercise groups showed significant improvements in SF-36 and MFIS, with no significant changes in mood indicators.
		CG=20	20/0	48.4 ± 9.8	3.1± 2.1	NR	Usual care	normal lifestyle		
Klefbeck et al., 2003 (40)	Sweden	IG=7	6/1	46 ± 3	7.5 ± 0.38	12 ± 4	Threshold inspiratory muscle trainer	10 weeks, two times/day,3 sets/time	FSS	Inspiratory muscle training significantly enhances inspiratory and expiratory muscle strength in severely disabled MS patients, with lasting effects, and no significant impact on lung function or FSS.
		CG=8	3/5	52.5 ± 5.75	8.0 ± 0.63	20 ± 5.75	Usual care	normal lifestyle		
Mostert et al., 2002 (36)	Switzerland	IG=13	10/3	45.23± 8.66	4.6± 1.2	11.2± 8.5	aerobic exercise	4 weeks, 30 minutes, £5 a week	FSS	Aerobic training significantly improves MS patients’ aerobic threshold, health perception, and exercise-related activity, with a tendency to alleviate MFIS.
		CG=13	11/2	43.9± 13.90	4.5± 1.9	12.6± 8.1	Usual care	normal lifestyle		

IG, intervention group; CG, control group; M, male; W, female; NR, not reported; TUG, Timed up and go time; BBS, Berg Balance Scale; 6MWT, 6-minute walk test; PSQI, Pittsburgh sleep quality index; FSS, Fatigue severity Scale; MFIS, Modified Fatigue Impact Scale; MSQOL-54, Quality of life-54 questionnaire; Sf-36, 36-item Short Form Health Survey.

### 3.3 Meta-analysis results

#### 3.3.1 Effects of breathing training on walking ability in patients with MS

**Figure 2:**
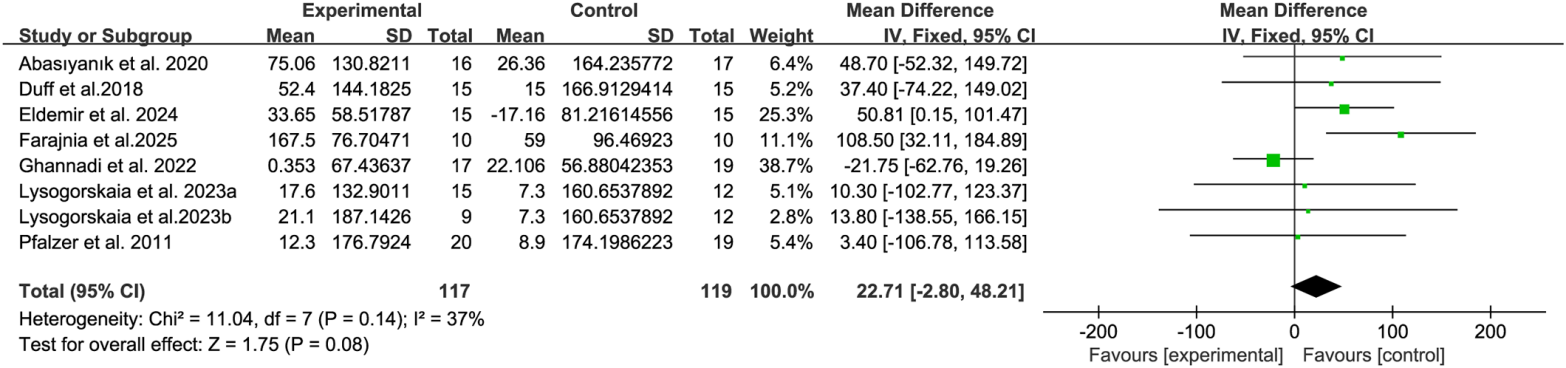
Meta-analysis of the effects of breathing training on 6-minute walk test (6WMT) in patients with MS.

6MWT was used to assess the walking ability of patients with MS. This study included 8 relevant studies that provided 6MWT data (15, 20, 32–34, 38, 39, 44–46, 48). The results show that compared with the control group, the breathing training (aerobic combined breathing training, breathing muscle training,aerobic exercise, Pilates, inspiratory muscle threshold training device,and yoga) do not show a significant improvement effect on the 6WMT score of patients with MS (WMD, 22.71; 95% CI, - 2.80 to 48.21, P = 0.08, I^2^ = 37%) (**Figure 2**).

#### 3.3.2 Effects of breathing training on balance ability in patients with MS

**Figure 3:**
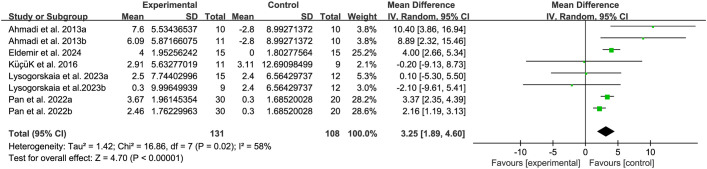
Meta-analysis of the effects of breathing training on Berg balance scale (BBS) in patients with MS.

**Figure 4:**
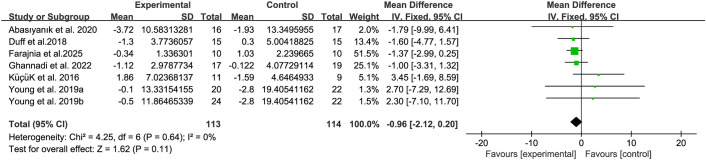
Meta-analysis of the effects of breathing training on timed up and go test (TUG) in patients with MS.

The balance of people with MS was detected by BBS and TUG, with 5 studies (34, 38, 39, 45, 48) providing BBS data and 5 studies (20, 32, 39, 44, 46) providing TUG data. The results show that compared with the control group, breathing training (yoga, Baduanjin, aerobic combined breathing training, aerobic training, and Pilates) has a significant effect on improving BBS (WMD, 3.25; 95% CI, 1.89 to 4.60, P < 0.00001, I^2^ = 58%) (**Figure 3**). However, compared with the control group, breathing training (aerobic training, Pilates, yoga), had no significant effect on the improvement of the TUG in patients with MS (WMD, -0.96; 95% CI, - 2.12 to 0.20, P = 0.11, I^2^ = 0%) (**Figure 4**).

#### 3.3.3 Effects of breathing training on quality of life in people with MS

**Figure 5:**
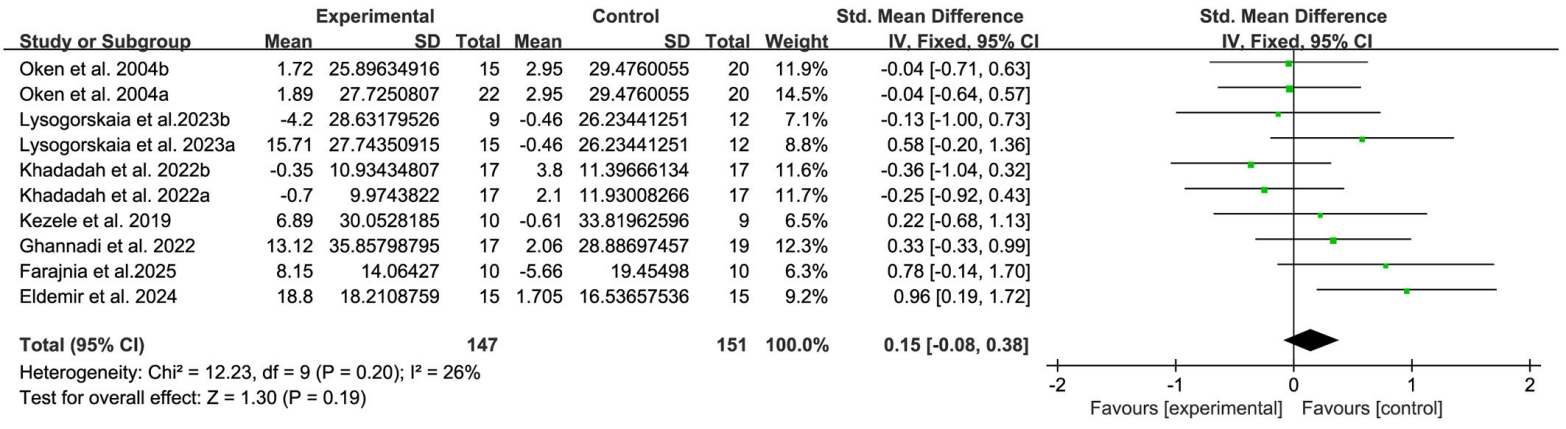
Meta-analysis of the effects of breathing training on quality of life in patients with MS.

The quality of life of patients with MS was evaluated using the SF-36 and the MSQOL-54. A total of 7 studies (20, 21, 35, 38, 42, 44, 45) providing data related to quality of life were included in this research. The results show that compared with the control group, breathing training had no significant effect on improving the quality of life scores of patients with MS (SMD, 0.15; 95%CI, -0.08 to 0.38, P= 0.19, I^2^ = 26%) (**Figure 5**).

#### 3.3.4 Effects of breathing training on fatigue in people with MS

**Figure 6:**
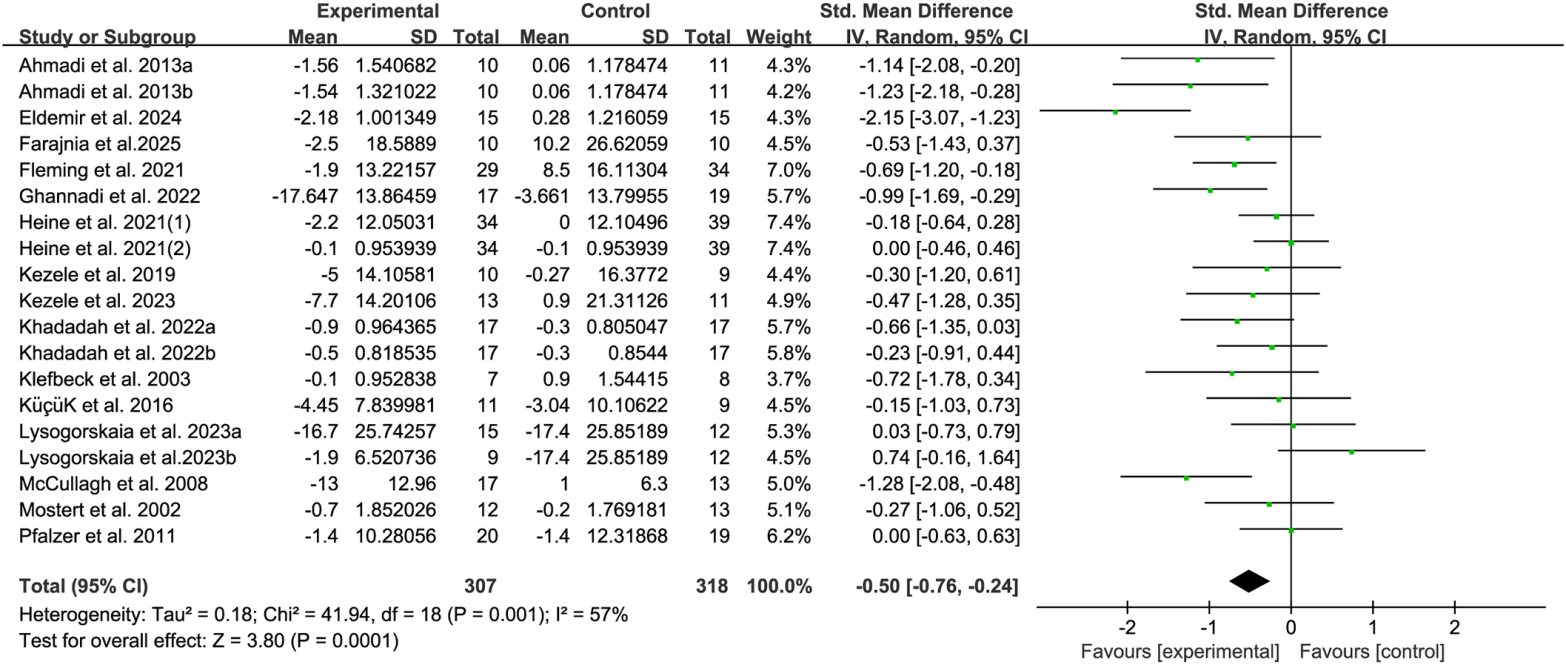
Meta-analysis of the effects of breathing training on fatigue in patients with MS.

The fatigue of people with MS was detected by FSS and MFIS. A total of 15 studies (20–22, 33, 36–45, 48) providing data related to fatigue were included. The results show that compared with the control group, breathing training (aerobic combined breathing training, breathing muscle training,aerobic exercise, Pilates, breathing muscle threshold training device, continuous positive airway pressure, and yoga) has a significant effect on improving the fatigue symptoms of patients with MS (SMD, -0.05; 95% CI, -0.76 to-0.24, P =0.0001, I^2^ = 57%) (**Figure 6**).

#### 3.3.5 Effect of breathing training on sleep quality in patients with MS

**Figure 7:**
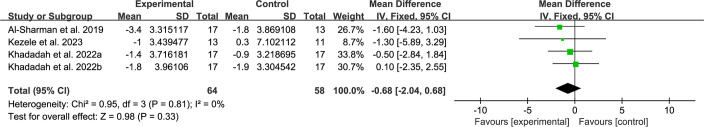
Meta-analysis of the effects of breathing training on Pittsburgh sleep quality index (PSQI) in patients with MS.

PSQI was used to assess the sleep quality of patients with MS. A total of 3 studies (21, 22, 47) providing data related to sleep quality were included in this research. The results show that compared with the control group, breathing training had no significant effect on the PSQI scores of patients (WMD, -0.68; 95%CI, − 2.04 to 0.68, P= 0.33, I^2^ = 0%) (**Figure 7**).

### 3.4 Subgroup analysis

#### 3.4.1 Quality of life

Based on the participants’ age, the methods of quality of life assessment, and the types of intervention, three different subgroup analyses were conducted respectively. The results showed that younger individuals demonstrated greater improvement in quality of life (young group, age < 45, SMD, 0.63; 95% CI, 0.24 to 1.01, P =0.001, I^2^ = 0%; middle-aged and older adults group, age ≥45, SMD, −0.12; 95% CI, −0.41 to 0.17, P =0.42, I^2^ = 0%) (**Supplementary Figure S1**).

Stratifying the analysis by type of quality of life detection, the improvement in quality of life scores remained not significant in MSQol-54 (SMD,0.17; 95% CI, −0.20 to 0.54, P = 0.37, I^2^ = 69%) and SF-36 (SMD, 0.14; 95% CI, −0.15 to 0.44, P = 0.35, I^2^ = 0%) (**Supplementary Figure S2**).

Furthermore, aerobic exercise (SMD, 0.16; 95% CI, −0.25 to 0.56, P = 0.46, I^2^ = 0%) and traditional breathing training (SMD, −0.08; 95% CI, −0.47 to 0.30, P =0.67, I^2^ = 17%) weren’t effective in improving quality of life in people with MS (**Supplementary Figure S3**).

#### 3.4.2 Fatigue

Three different subgroup analyses were conducted based on the participants’ age, fatigue assessment type, and intervention type. The results showed that younger individuals demonstrated greater improvement in fatigue (young group, age < 45, SMD, −0.76; 95% CI, −1.23 to −0.30, P =0.001, I^2^ = 73%; middle-aged and older adult group, age ≥ 45, SMD, −0.31; 95% CI, −0.57 to −0.06, P =0.01, I^2^ = 12%) (**Supplementary Figure S4**).

Stratifying the analysis by type of fatigue detection, the improvement in fatigue scores remained significant in FSS (SMD, −0.64; 95% CI, −1.07 to −0.20, P = 0.004, I^2^ = 67%) and MFIS (SMD, −0.41; 95% CI, −0.73 to −0.09, P = 0.01, I^2^ = 48%) (**Supplementary Figure S5**).

In addition, aerobic exercise (SMD, −0.35; 95% CI, −0.69 to 0.00, P = 0.05, I^2^ = 51%) and Pilates (SMD, −0.97; 95% CI, −1.97 to 0.03, P =0.06, I^2^ = 81%) weren’t effective in improving fatigue in people with MS. However, traditional breathing training (SMD, −0.48; 95% CI, -0.85 to- 0.11, P = 0.01, I^2^ = 24%) can effectively improve the fatigue in people with MS (**Supplementary Figure S6**).

### 3.5 Sensitivity analysis

The differences were found by eliminating the included literatures one by one. After the literature of Eldemir et al. (43) was excluded from the studies with fatigue as the outcome index, the heterogeneity was reduced from 57% to 40%. The heterogeneity did not change much after excluding other literatures, suggesting that this study was the main source of heterogeneity in this study (**Supplementary Figure S7**).

### 3.6 Risk of bias

The quality of 21 included studies was evaluated independently according to the Cochrane RCT bias risk assessment tool (**Supplementary Figures S8**, **S9**). According to the PEDro scale evaluation results (**Supplementary Table S1**), the average quality score of the 21 articles was 6.24, indicating medium to high quality.

### 3.7 Publication bias

Funnel plot analysis was performed using BBS, 6WMT, TUG, Figure, quality of life and PSQI as indicators for the included literature. There was no obvious asymmetry in the funnel plot, suggesting that publication bias was small (**Supplementary Figure S10**).

## 4 Discussion

As a non-pharmacological intervention, breathing training has been widely used in the field of nervous system rehabilitation in recent years (49). An increasing number of studies have confirmed the impact of breathing training on the balance and quality of life of stroke patients (50–52). This systematic review and meta-analysis evaluated the effects of breathing training on walking ability and quality of life in patients with MS. The results showed that the effects of breathing training on different functional indexes in patients with MS were different. Subgroup analysis showed that older age was associated with less significant improvement in fatigue.

Breathing training did not improve the 6WMT, which is consistent with previous studies (20), because 6WMT mainly measures the ability of continuous walking, which has high requirements for patients’ cardiopulmonary function and overall function (53). This suggests that 6WMT may be less responsive to the effects of breathing training alone, as it reflects more comprehensive physical capacity. Moreover, the included studies lasted for 8–12 weeks, which may not be sufficient to cause significant changes in 6WMT (54, 55). In addition, different EDSS grades may have different responses to the same intervention, which could also contribute to the lack of significant improvement in this indicator.

In contrast, the research results show that there was a significant improvement in the BBS score in terms of balance, which is consistent with the findings of a previous study (48). This study demonstrated that breathing training is effective in improving balance in patients with MS. The improvement effect of breathing training on balance ability involves multiple physiological mechanisms, which together form the basis of its therapeutic effect. The close correlation between breathing muscle function and postural control is one of the key mechanisms by which breathing training improves balance. The diaphragm, as the main inspiratory muscle, participates in both breathing and postural regulation. Through enhancing the strength of the diaphragm and auxiliary breathing muscles, breathing training can significantly improve thoracic cage stability and thereby enhance trunk control ability (56, 57). Additionally, the core stability mechanism explains how breathing training indirectly affects balance ability. The diaphragm, pelvic floor muscles, and abdominal muscles together constitute the “core” system. Breathing training can enhance the muscle strength and coordination of these muscles. Through targeted breathing training, a physiological chain of “abdominal pressure regulation-trunk stability” is established (52, 58). In recent years, several studies examining the effect of breathing exercises on trunk control and balance (56, 58, 59) have also supported these mechanisms.

However, there was no significant improvement in the timed up and go test (TUG), which was consistent with the results of previous studies (15). This is because the BBS mainly assesses static and dynamic balance (60), and more reflects postural control and small-scale movement ability, making it sensitive to small early changes induced by breathing training. In contrast, the TUG is a comprehensive assessment of daily activities such as standing up, walking, and turning (61), and also requires greater lower limb strength and coordination—factors that may not be sufficiently addressed by breathing training alone. This further suggests that while BBS is sensitive to subtle improvements, TUG (like 6WMT) requires larger functional changes to be reflected (54, 55).

The results suggest that for walking ability-related indicators, simple breathing training may be considered for patients with MS with balance dysfunction, whereas for those with walking impairment, lower limb strength training should be combined with breathing training. A stepwise rehabilitation approach is recommended: start with simple breathing exercises to improve balance, then introduce more complex walking training methods. Future research could explore differences in breathing training effects across EDSS grades and dynamically assess the synergistic activation patterns of breathing and walking muscle groups using surface electromyography (SEMG) (61).

The above analysis results indicate that there was no significant improvement in quality of life as measured by the SF-36 and MSQOL-54, which is consistent with previous studies (21). This is because the SF-36 and MSQOL encompass broader dimensions such as social functioning and emotional roles, which are influenced by multiple factors beyond respiratory function (57). Quality of life is a comprehensive evaluation standard covering physiological, psychological, social, and environmental dimensions, and while breathing training can improve breathing function-optimizing oxygenation to extend exercise duration, enhancing exercise endurance and core stability to reduce fall risk, prolong social participation time, and minimize fatigue-related limitations in daily life (62, 63). These effects may not yet be sufficient to translate into measurable improvements in the multi-dimensional SF-36 and MSQOL-54 within the study timeframe.

By comparison, breathing training significantly improved the fatigue condition of patients with MS as measured by the FSS and MFIS, consistent with previous research findings (22). This suggests that fatigue alleviation is a more direct and sensitive outcome of improved breathing function. Subgroup analysis further revealed that conventional breathing training (MD=-2.64) was more effective than aerobic breathing training (MD=-0.05) in reducing fatigue, indicating that MS patients with severe breathing dysfunction may benefit from first undergoing specialized breathing training before transitioning to comprehensive training programs aligning with the aforementioned strategy of gradual rehabilitation implementation (64).

Consistently, the PSQI also showed no significant improvement with breathing training, which is consistent with previous studies (21, 60). This is likely because sleep quality, as assessed by the PSQI, is influenced by non-respiratory factors such as pain and psychological status (53).

This study identifies several key methodological challenges in current research on breathing training for patients with MS. Firstly, significant heterogeneity exists in the assessment tools used across different studies, reflecting the lack of standardized efficacy evaluation systems in this field. We strongly recommend that future studies adopt internationally recognized Core Outcome Sets to improve the comparability of research findings and their clinical translational value. Secondly, research in this area remains in the exploratory clinical stage, with generally small sample sizes limiting statistical power. Therefore, we advocate for multicenter collaborative studies to pool resources and obtain sufficient sample sizes, thereby providing more reliable effect size estimates. Regarding control of confounding factors, although we strictly included studies with matched baseline medications, the lack of information on medication adjustments during the intervention period may still affect result interpretation. Thus, future randomized controlled trials should: (1) meticulously document concomitant medication use; (2) establish medication stability as an inclusion criterion; and (3) consider incorporating medication washout periods to more accurately assess the independent therapeutic effects of breathing training. Particularly noteworthy is that disease severity (EDSS stratification) may be an important effect modifier. However, the current analysis was limited by: (1) 20% of studies not reporting EDSS data; (2) inconsistencies in assessment tools among existing data. These methodological gaps suggest that establishing standardized disease staging reporting guidelines should be a priority for future research. We recommend that subsequent studies systematically examine response differences to breathing training across EDSS strata to facilitate the development of personalized rehabilitation protocols. Despite these limitations, this meta-analysis is the first to comprehensively evaluate the potential benefits of breathing training for the functional and quality of life aspects of patients with MS, providing preliminary evidence for its clinical application. The results of this study suggest support for incorporating breathing training into a multimodal treatment regimen for MS as a complementary treatment to drug therapy. In the future, more high-quality and methodologically rigorous research are needed to optimize the treatment plan for patients with MS, clarify its mechanism of action, and evaluate the long-term effect of breathing training. Clinicians may need to develop an individualized breathing training program for the specific dysfunction of patients with MS and integrate it with existing disease management programs. With the development of modern rehabilitation medicine, breathing training is expected to become one of the means to improve the functional prognosis of patients with MS.

## 5 Strengths and limitations of this systematic review

This study, as the first meta-analysis systematically evaluating the impact of breathing training on both walking capacity and quality of life in patients with MS, fills a critical gap in the current body of relevant evidence. Strictly adhering to the PRISMA guidelines for literature screening, it effectively reduces selection bias and reporting bias through dual-independent review and quality assessment (such as the application of the PEDro scale). Meanwhile, by comprehensively analyzing outcome indicators from multiple dimensions including walking capacity and quality of life, it provides a basis for formulating comprehensive rehabilitation strategies. Additionally, this study further enhances the relevance and applicability of the results by conducting multiple subgroup analyses.

However, this study still has certain limitations. Firstly, there is a high degree of heterogeneity in the breathing training protocols of the included studies, including aspects such as training types, intensity, frequency, single-session duration, and intervention cycles, making it difficult to draw clear conclusions regarding the dose-response relationship. Secondly, the assessment criteria adopted in various studies (such as lung function and balance ability) are inconsistent, and there is a lack of unified indicators for measuring the dose-response relationship. Thirdly, due to the nature of breathing training, it is impossible to blind participants, which may introduce performance bias. Fourthly, the limited availability of long term follow-up data makes it impossible to analyze the sustainability of the intervention effects. Consequently, due to the aforementioned methodological differences, this meta-analysis faces difficulties in directly comparing the effects of different intervention approaches.

In summary, the optimal dosage of breathing training and its long term effects still require further research. Future studies should standardize training parameters (including intervention type, intensity, frequency, single session duration, and intervention cycle) and incorporate long term follow-up designs.

## 6 Conclusion

This study systematically evaluated the effects of breathing training on functional rehabilitation in patients with multiple sclerosis (MS) through meta-analysis. In terms of walking function, the balance function was significantly improved, but there was no significant change in functional mobility (TUG, 6MWT). In terms of quality of life, fatigue symptoms were significantly relieved, however, the global quality of life assessed by SF-36 and MSQOL-54 did not reach the significance threshold, and there was no significant change in sleep quality assessed by PSQI.

In conclusion, breathing training can effectively improve the balance function and relieve the symptoms of fatigue in patients with MS, but the effect on functional mobility, overall quality of life and sleep quality has not been supported by evidence. Future research should establish a standardized breathing training program, extend the intervention period to verify the long-term benefits, and explore the differences in the efficacy of different subgroups (such as disease stage and disability degree) to further verify its long-term benefits on comprehensive functional rehabilitation.

## Data Availability

The original contributions presented in the study are included in the article/**Supplementary Material**. Further inquiries can be directed to the corresponding author.
