# Supplementary material for: Effects of breathing training on walking ability and quality of life in patients with multiple sclerosis: systematic review and meta-analysis of randomized controlled trials
Source: Front Immunol. 2025 Aug 29;16:1643938. doi: 10.3389/fimmu.2025.1643938 (PMC12425779; doi:10.3389/fimmu.2025.1643938)
Supplement: Supplementary file 1 [file DataSheet1.pdf]

Supplementary Material

1 Supplementary Data

1.1 Details of the search

PubMed

#	Search terms	Results
1	((((("Multiple Sclerosis"[Mesh]) OR (Sclerosis, Multiple)) OR (MS (Multiple Sclerosis))) OR (Sclerosis, Disseminated)) OR (Disseminated Sclerosis)) OR (Multiple Sclerosis, Acute Fulminating)	121,229
2	((((((((((((((((((((("Breathing Exercises"[Mesh]) OR (Exercise, Breathing)) OR (Respiratory Muscle Training)) OR (Muscle Training, Respiratory)) OR (Training, Respiratory Muscle)) OR (exercise therapy)) OR (Respiration)) OR (breathing exercise)) OR (Inspiration)) OR (Expiration)) OR (inspiratory muscle training)) OR (expiratory muscle strength training)) OR (respiratory therapy)) OR (breathing control)) OR (breath)) OR (breath)) OR (pranayama)) OR (yoga)) OR (Strength training)) OR (Aerobic training)) OR (Tai Chi)) OR (Pilates)) OR (cycle ergometer)	2,343,720
3	(#1) AND (#2)	7,323
4	(((Randomized controlled trial) OR (randomized)) OR (placebo)) OR (RCT)	1,754,620
5	(#3) AND (#4)	1,159

Embase

#	Search terms	Results
1	'multiple sclerosis'/exp OR 'multiple sclerosis'	204,581
2	'Sclerosis, Multiple':ab,ti OR 'MS (Multiple Sclerosis)':ab,ti OR 'Sclerosis, Disseminated':ab,ti OR 'Disseminated Sclerosis':ab,ti OR 'Multiple Sclerosis, Acute Fulminating':ab,ti	1,181
3	#1OR#2	204,691

4	'breathing exercise'/exp OR 'breathing exercise'	1,4619
5	'Exercise, Breathing':ab,ti OR 'Respiratory Muscle Training':ab,ti OR 'Muscle Training, Respiratory':ab,ti OR 'Training, Respiratory Muscle':ab,ti OR 'exercise therapy ':ab,ti OR 'Respiration':ab,ti OR ' breathing exercise ':ab,ti OR 'Inspiration':ab,ti OR 'Expiration':ab,ti OR 'inspiratory muscle training':ab,ti OR 'expiratory muscle strength training':ab,ti OR 'respiratory therapy':ab,ti OR 'breathing control':ab,ti OR 'breath':ab,ti OR 'exercise':ab,ti OR 'pranayama':ab,ti OR 'yoga':ab,ti OR 'Strength training':ab,ti OR 'Aerobic training':ab,ti OR 'cycle ergometer':ab,ti OR 'Tai Chi':ab,ti OR 'Pilates':ab,ti	749,938
6	#4 OR #5	758,306
7	#3 AND #6	**1.1.1** 4,168
8	'randomized controlled trial':ab,ti OR 'randomized':ab,ti OR 'placebo':ab,ti OR 'rct':ab,ti	1,060
9	#7 AND #8	1,004

Web of Science

#	Search terms	Results
1	TS=(Multiple Sclerosis OR Sclerosis, Multiple OR MS (Multiple Sclerosis) OR Sclerosis, Disseminated OR Disseminated Sclerosis OR Multiple Sclerosis, Acute Fulminating)	225,808
2	TS=(Breathing Exercises OR Exercise, Breathing OR Respiratory Muscle Training OR Muscle Training, Respiratory OR Training, Respiratory Muscle OR exercise therapy OR Respiration OR breathing exercise OR Inspiration OR Expiration OR inspiratory muscle training OR expiratory muscle strength training OR respiratory therapy OR breathing control OR breath OR exercise OR pranayama OR yoga OR Strength training OR Aerobic training OR cycle ergometer OR Tai Chi OR Pilates)	3,037,314
3	#1 AND #2	10,898
4	TS=(Randomized controlled trial OR randomized OR placebo OR RCT)	1,818,543
5	#3 AND #4	1,971

Cochrane Library

#	Search terms	Results
1	MeSH descriptor: [Multiple Sclerosis] explode all trees	5,405
2	(Sclerosis, Multiple):ti,ab,kw OR (MS (Multiple Sclerosis)):ti,ab,kw OR (Sclerosis, Disseminated):ti,ab,kw OR (Disseminated Sclerosis):ti,ab,kw OR (Multiple Sclerosis, Acute Fulminating):ti,ab,kw	13,650
3	#1 OR #2	13,650
4	MeSH descriptor: [Breathing Exercises] explode all trees	1448
5	(Exercise, Breathing):ti,ab,kw OR (Respiratory Muscle Training):ti,ab,kw OR (Muscle Training, Respiratory):ti,ab,kw OR (Training, Respiratory Muscle):ti,ab,kw OR (exercise therapy):ti,ab,kw OR (Respiration):ti,ab,kw OR (breathing exercise):ti,ab,kw OR (Inspiration):ti,ab,kw OR (Expiration):ti,ab,kw OR (inspiratory muscle training):ti,ab,kw OR (expiratory muscle strength training):ti,ab,kw OR (respiratory therapy):ti,ab,kw OR (breathing control):ti,ab,kw OR (breath):ti,ab,kw OR (exercise):ti,ab,kw OR (pranayama):ti,ab,kw OR (yoga):ti,ab,kw OR (Strength training):ti,ab,kw OR (Aerobic training):ti,ab,kw OR (cycle ergometer):ti,ab,kw OR (Tai Chi):ti,ab,kw OR (Pilates):ti,ab,kw	226,784
6	#4 OR #5	226,873
7	#3 AND #6	1,961
8	(Randomized controlled trial):ti,ab,kw OR (randomized):ti,ab,kw OR (placebo):ti,ab,kw OR (RCT):ti,ab,kw	1,360,929
9	#7 AND #8	1,430
10	Trials	1,386

2 Supplementary Figures and Tables

2.1 Supplementary Figures

Study or Subgroup	Experimental Mean	Experimental SD	Experimental Total	Control Mean	Control SD	Control Total	Weight	Std. Mean Difference IV, Fixed, 95% CI
**6.1.1 age＜45**								
Eldemir et al. 2024	18.8	18.21088	15	1.705	16.53658	15	9.2%	0.96 [0.19, 1.72]
Farajnia et al.2025	8.15	14.06427	10	-5.66	19.45498	10	6.3%	0.78 [-0.14, 1.70]
Ghannadi et al. 2022	13.12	35.85799	17	2.06	28.88697	19	12.3%	0.33 [-0.33, 0.99]
Lysogorskaia et al. 2023a	15.71	27.74351	15	-0.46	26.23441	12	8.8%	0.58 [-0.20, 1.36]
**Subtotal (95% CI)**			**57**			**56**	**36.6%**	**0.63 [0.24, 1.01]**
Heterogeneity: Chi² = 1.60, df = 3 (P = 0.66); I² = 0%								
Test for overall effect: Z = 3.22 (P = 0.001)								
**6.1.2 age≥45**								
Kezele et al. 2019	6.89	30.05282	10	-0.61	33.81963	9	6.5%	0.22 [-0.68, 1.13]
Khadadah et al. 2022a	-0.7	9.974382	17	2.1	11.93008	17	11.7%	-0.25 [-0.92, 0.43]
Khadadah et al. 2022b	-0.35	10.93435	17	3.8	11.39666	17	11.6%	-0.36 [-1.04, 0.32]
Lysogorskaia et al.2023b	-4.2	28.6318	9	-0.46	26.23441	12	7.1%	-0.13 [-1.00, 0.73]
Oken et al. 2004a	1.89	27.72508	22	2.95	29.47601	20	14.5%	-0.04 [-0.64, 0.57]
Oken et al. 2004b	1.72	25.89635	15	2.95	29.47601	20	11.9%	-0.04 [-0.71, 0.63]
**Subtotal (95% CI)**			**90**			**95**	**63.4%**	**-0.12 [-0.41, 0.17]**
Heterogeneity: Chi² = 1.31, df = 5 (P = 0.93); I² = 0%								
Test for overall effect: Z = 0.81 (P = 0.42)								
**Total (95% CI)**			**147**			**151**	**100.0%**	**0.15 [-0.08, 0.38]**
Heterogeneity: Chi² = 12.23, df = 9 (P = 0.20); I² = 26%								
Test for overall effect: Z = 1.30 (P = 0.19)								
Test for subgroup differences: Chi² = 9.32, df = 1 (P = 0.002), I² = 89.3%								

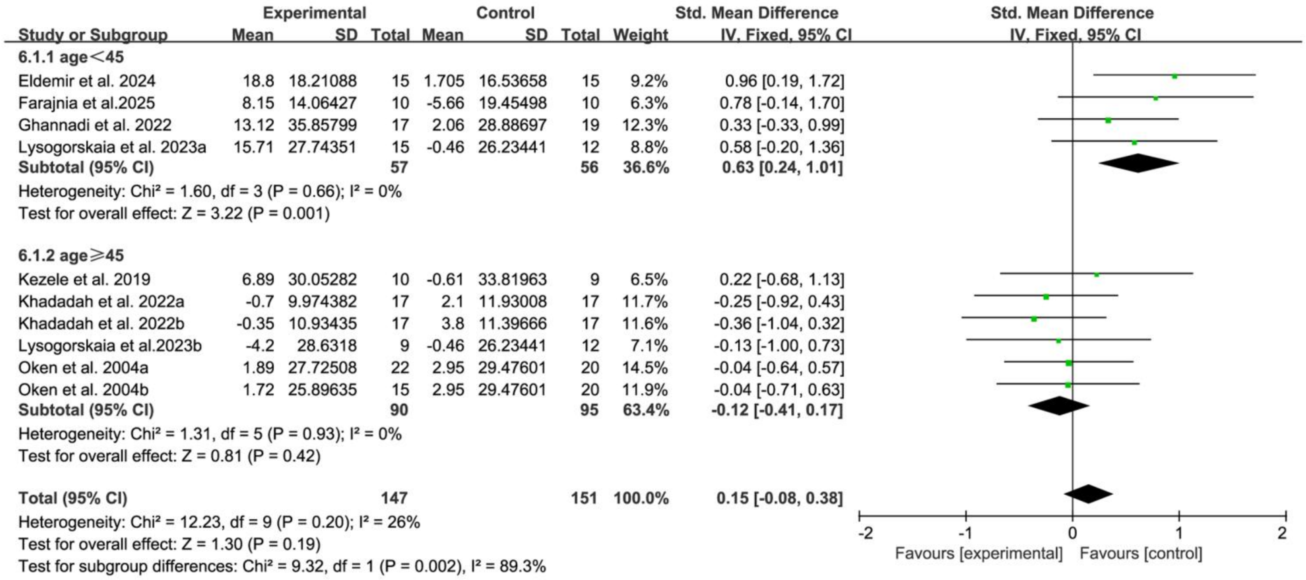

Supplementary Figure 1. Meta-analysis of the effect of participants′ age on quality of life in MS patients.

Study or Subgroup	Experimental Mean	Experimental SD	Experimental Total	Control Mean	Control SD	Control Total	Weight	Std. Mean Difference IV, Fixed, 95% CI
**5.1.1 MsQol-54**								
Eldemir et al. 2024	18.8	18.21088	15	1.705	16.53658	15	9.2%	0.96 [0.19, 1.72]
Farajnia et al.2025	8.15	14.06427	10	-5.66	19.45498	10	6.3%	0.78 [-0.14, 1.70]
Khadadah et al. 2022a	-0.7	9.974382	17	2.1	11.93008	17	11.7%	-0.25 [-0.92, 0.43]
Khadadah et al. 2022b	-0.35	10.93435	17	3.8	11.39666	17	11.6%	-0.36 [-1.04, 0.32]
**Subtotal (95% CI)**			**59**			**59**	**38.8%**	**0.17 [-0.20, 0.54]**
Heterogeneity: Chi² = 9.64, df = 3 (P = 0.02); I² = 69%								
Test for overall effect: Z = 0.90 (P = 0.37)								
**5.1.2 SF-36**								
Ghannadi et al. 2022	13.12	35.85799	17	2.06	28.88697	19	12.3%	0.33 [-0.33, 0.99]
Kezele et al. 2019	6.89	30.05282	10	-0.61	33.81963	9	6.5%	0.22 [-0.68, 1.13]
Lysogorskaia et al. 2023a	15.71	27.74351	15	-0.46	26.23441	12	8.8%	0.58 [-0.20, 1.36]
Lysogorskaia et al.2023b	-4.2	28.6318	9	-0.46	26.23441	12	7.1%	-0.13 [-1.00, 0.73]
Oken et al. 2004a	1.89	27.72508	22	2.95	29.47601	20	14.5%	-0.04 [-0.64, 0.57]
Oken et al. 2004b	1.72	25.89635	15	2.95	29.47601	20	11.9%	-0.04 [-0.71, 0.63]
**Subtotal (95% CI)**			**88**			**92**	**61.2%**	**0.14 [-0.15, 0.44]**
Heterogeneity: Chi² = 2.58, df = 5 (P = 0.76); I² = 0%								
Test for overall effect: Z = 0.94 (P = 0.35)								
**Total (95% CI)**			**147**			**151**	**100.0%**	**0.15 [-0.08, 0.38]**
Heterogeneity: Chi² = 12.23, df = 9 (P = 0.20); I² = 26%								
Test for overall effect: Z = 1.30 (P = 0.19)								
Test for subgroup differences: Chi² = 0.01, df = 1 (P = 0.91), I² = 0%								

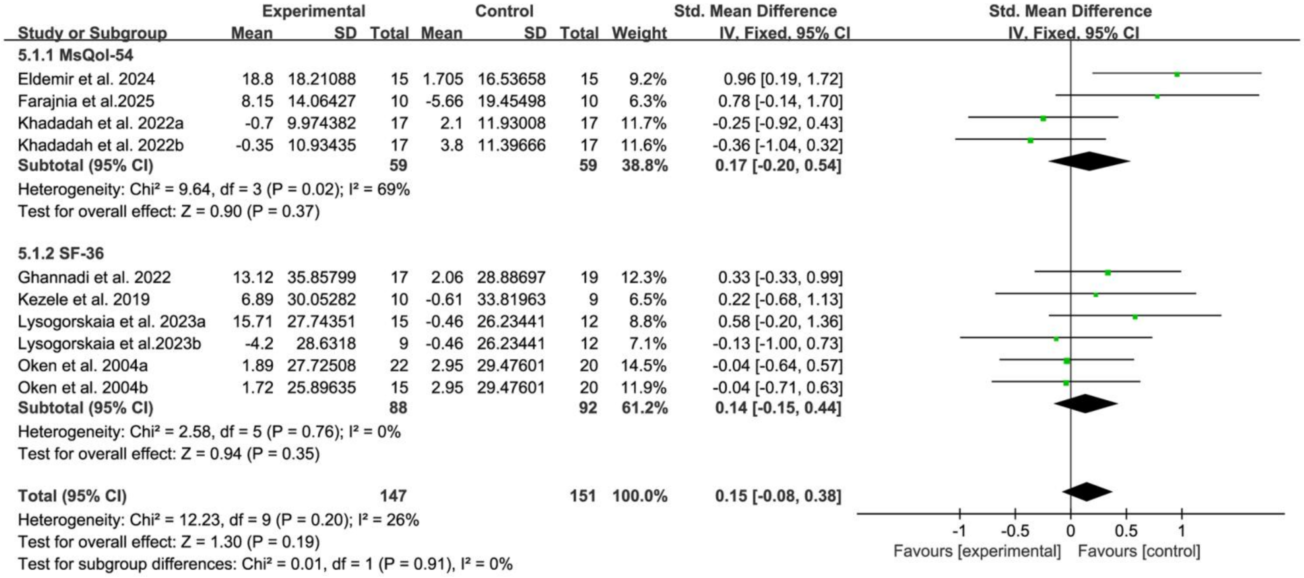

Supplementary Figure 2. Meta-analysis of the effect of type of quality of life test on quality of life in MS patients on quality of life in MS patients.

Study or Subgroup	Experimental Mean	Experimental SD	Experimental Total	Control Mean	Control SD	Control Total	Weight	Std. Mean Difference IV, Fixed, 95% CI
**7.1.1 aerobic training**								
Farajnia et al.2025	8.15	14.06427	10	-5.66	19.45498	10	9.4%	0.78 [-0.14, 1.70]
Kezele et al. 2019	6.89	30.05282	10	-0.61	33.81963	9	9.7%	0.22 [-0.68, 1.13]
Lysogorskaia et al.2023b	-4.2	28.6318	9	-0.46	26.23441	12	10.6%	-0.13 [-1.00, 0.73]
Oken et al. 2004b	1.72	25.89635	15	2.95	29.47601	20	17.6%	-0.04 [-0.71, 0.63]
**Subtotal (95% CI)**			**44**			**51**	**47.3%**	**0.16 [-0.25, 0.56]**
Heterogeneity: Chi² = 2.56, df = 3 (P = 0.46); I² = 0%								
Test for overall effect: Z = 0.75 (P = 0.46)								
**7.1.2 Traditional breathing training**								
Ghannadi et al. 2022	13.12	35.85799	17	2.06	28.88697	19	18.2%	0.33 [-0.33, 0.99]
Khadadah et al. 2022a	-0.7	9.974382	17	2.1	11.93008	17	17.3%	-0.25 [-0.92, 0.43]
Khadadah et al. 2022b	-0.35	10.93435	17	3.8	11.39666	17	17.2%	-0.36 [-1.04, 0.32]
**Subtotal (95% CI)**			**51**			**53**	**52.7%**	**-0.08 [-0.47, 0.30]**
Heterogeneity: Chi² = 2.42, df = 2 (P = 0.30); I² = 17%								
Test for overall effect: Z = 0.43 (P = 0.67)								
**Total (95% CI)**			**95**			**104**	**100.0%**	**0.03 [-0.25, 0.31]**
Heterogeneity: Chi² = 5.68, df = 6 (P = 0.46); I² = 0%								
Test for overall effect: Z = 0.20 (P = 0.84)								
Test for subgroup differences: Chi² = 0.70, df = 1 (P = 0.40), I² = 0%								

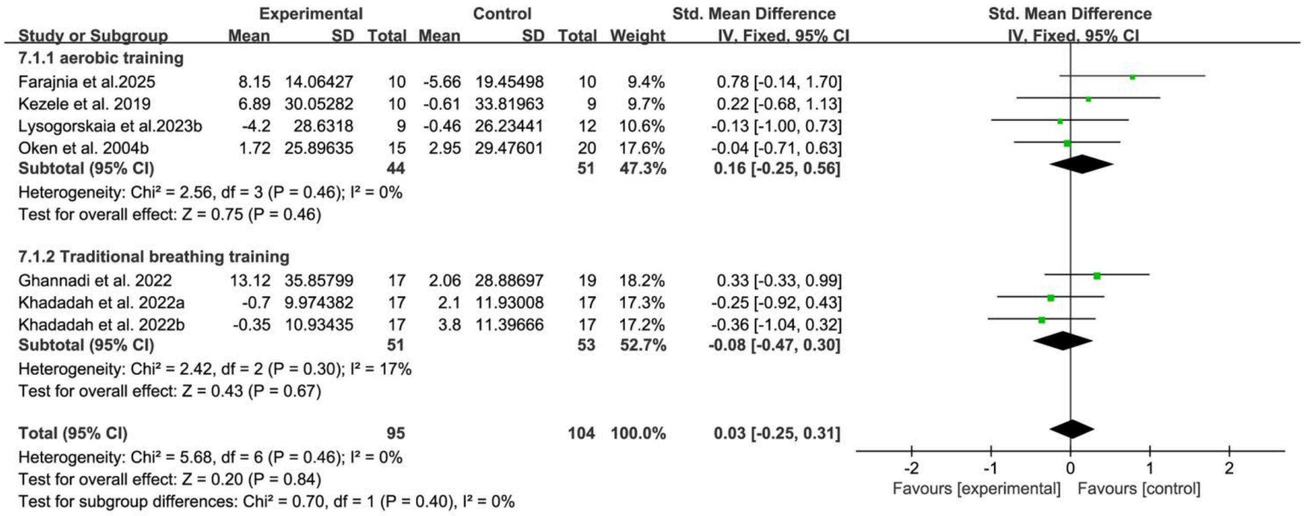

Supplementary Figure 3. Meta-analysis of the effect of type of intervention on quality of life in MS patients on quality of life in MS patients.

Study or Subgroup	Experimental Mean	Experimental SD	Experimental Total	Control Mean	Control SD	Control Total	Weight	Std. Mean Difference IV, Random, 95% CI
**3.1.1 age<45**								
Ahmadi et al. 2013a	-1.56	1.540682	10	0.06	1.178474	11	4.3%	-1.14 [-2.08, -0.20]
Ahmadi et al. 2013b	-1.54	1.321022	10	0.06	1.178474	11	4.2%	-1.23 [-2.18, -0.28]
Eldemir et al. 2024	-2.18	1.001349	15	0.28	1.216059	15	4.3%	-2.15 [-3.07, -1.23]
Farajnia et al.2025	-2.5	18.5889	10	10.2	26.62059	10	4.5%	-0.53 [-1.43, 0.37]
Ghannadi et al. 2022	-17.647	13.86459	17	-3.661	13.79955	19	5.7%	-0.99 [-1.69, -0.29]
Heine et al. 2021(1)	-2.2	12.05031	34	0	12.10496	39	7.4%	-0.18 [-0.64, 0.28]
Heine et al. 2021(2)	-0.1	0.953939	34	-0.1	0.953939	39	7.4%	0.00 [-0.46, 0.46]
Lysogorskaia et al. 2023a	-16.7	25.74257	15	-17.4	25.85189	12	5.3%	0.03 [-0.73, 0.79]
McCullagh et al. 2008	-13	12.96	17	1	6.3	13	5.0%	-1.28 [-2.08, -0.48]
**Subtotal (95% CI)**			**162**			**169**	**48.1%**	**-0.76 [-1.23, -0.30]**
Heterogeneity: Tau² = 0.35; Chi² = 29.92, df = 8 (P = 0.0002); I² = 73%								
Test for overall effect: Z = 3.24 (P = 0.001)								
**3.1.2 age⩾45**								
Fleming et al. 2021	-1.9	13.22157	29	8.5	16.11304	34	7.0%	-0.69 [-1.20, -0.18]
Kezele et al. 2019	-5	14.10581	10	-0.27	16.3772	9	4.4%	-0.30 [-1.20, 0.61]
Kezele et al. 2023	-7.7	14.20106	13	0.9	21.31126	11	4.9%	-0.47 [-1.28, 0.35]
Khadadah et al. 2022a	-0.9	0.964365	17	-0.3	0.805047	17	5.7%	-0.66 [-1.35, 0.03]
Khadadah et al. 2022b	-0.5	0.818535	17	-0.3	0.8544	17	5.8%	-0.23 [-0.91, 0.44]
Klefbeck et al. 2003	-0.1	0.952838	7	0.9	1.54415	8	3.7%	-0.72 [-1.78, 0.34]
KüçüK et al. 2016	-4.45	7.839981	11	-3.04	10.10622	9	4.5%	-0.15 [-1.03, 0.73]
Lysogorskaia et al.2023b	-1.9	6.520736	9	-17.4	25.85189	12	4.5%	0.74 [-0.16, 1.64]
Mostert et al. 2002	-0.7	1.852026	12	-0.2	1.769181	13	5.1%	-0.27 [-1.06, 0.52]
Pfalzer et al. 2011	-1.4	10.28056	20	-1.4	12.31868	19	6.2%	0.00 [-0.63, 0.63]
**Subtotal (95% CI)**			**145**			**149**	**51.9%**	**-0.31 [-0.57, -0.06]**
Heterogeneity: Tau² = 0.02; Chi² = 10.18, df = 9 (P = 0.34); I² = 12%								
Test for overall effect: Z = 2.46 (P = 0.01)								
**Total (95% CI)**			**307**			**318**	**100.0%**	**-0.50 [-0.76, -0.24]**
Heterogeneity: Tau² = 0.18; Chi² = 41.94, df = 18 (P = 0.001); I² = 57%								
Test for overall effect: Z = 3.80 (P = 0.0001)								
Test for subgroup differences: Chi² = 2.82, df = 1 (P = 0.09), I² = 64.5%								

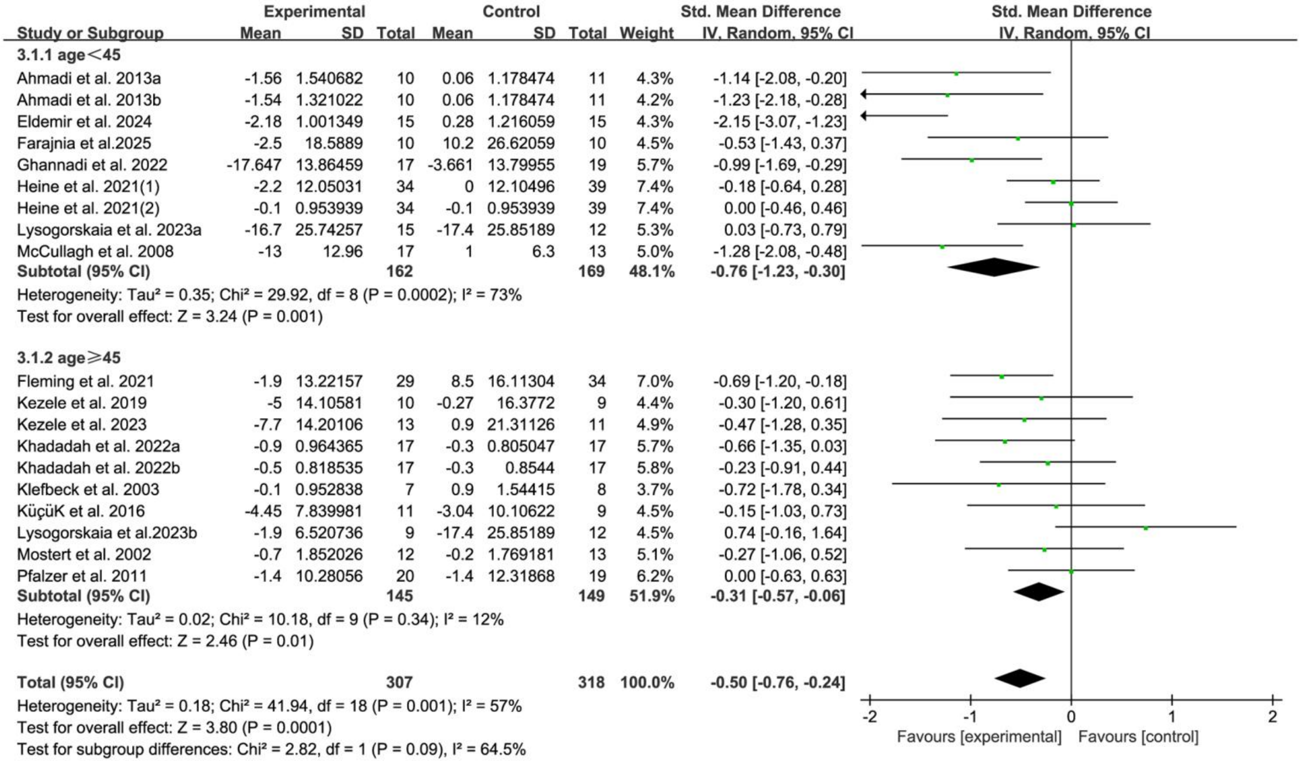

Supplementary Figure 4. Meta-analysis of the effect of participants′ age on fatigue in MS patients.

Study or Subgroup	Experimental Mean	Experimental SD	Experimental Total	Control Mean	Control SD	Control Total	Weight	Std. Mean Difference IV, Random, 95% CI
**2.1.1 MFIS**								
Farajnia et al.2025	-2.5	18.5889	10	10.2	26.62059	10	4.5%	-0.53 [-1.43, 0.37]
Fleming et al. 2021	-1.9	13.22157328	29	8.5	16.1130382	34	7.0%	-0.69 [-1.20, -0.18]
Ghannadi et al. 2022	-17.647	13.86459372	17	-3.661	13.79954829	19	5.7%	-0.99 [-1.69, -0.29]
Heine et al. 2021(1)	-2.2	12.0503112	34	0	12.10495766	39	7.4%	-0.18 [-0.64, 0.28]
Kezele et al. 2019	-5	14.10581086	10	-0.27	16.37720062	9	4.4%	-0.30 [-1.20, 0.61]
Kezele et al. 2023	-7.7	14.2010563	13	0.9	21.31126463	11	4.9%	-0.47 [-1.28, 0.35]
KüçüK et al. 2016	-4.45	7.83998087	11	-3.04	10.10621591	9	4.5%	-0.15 [-1.03, 0.73]
Lysogorskaia et al. 2023a	-16.7	25.74257174	15	-17.4	25.85188581	12	5.3%	0.03 [-0.73, 0.79]
Lysogorskaia et al.2023b	-1.9	6.52073616	9	-17.4	25.85188581	12	4.5%	0.74 [-0.16, 1.64]
McCullagh et al. 2008	-13	12.96	17	1	6.3	13	5.0%	-1.28 [-2.08, -0.48]
**Subtotal (95% CI)**			**165**			**168**	**53.3%**	**-0.41 [-0.73, -0.09]**
Heterogeneity: Tau² = 0.12; Chi² = 17.33, df = 9 (P = 0.04); I² = 48%								
Test for overall effect: Z = 2.49 (P = 0.01)								
**2.1.2 FSS**								
Ahmadi et al. 2013a	-1.56	1.54068167	10	0.06	1.17847359	11	4.3%	-1.14 [-2.08, -0.20]
Ahmadi et al. 2013b	-1.54	1.32102233	10	0.06	1.17847359	11	4.2%	-1.23 [-2.18, -0.28]
Eldemir et al. 2024	-2.18	1.00134909	15	0.28	1.21605921	15	4.3%	-2.15 [-3.07, -1.23]
Heine et al. 2021(2)	-0.1	0.9539392	34	-0.1	0.9539392	39	7.4%	0.00 [-0.46, 0.46]
Khadadah et al. 2022a	-0.9	0.96436508	17	-0.3	0.80504658	17	5.7%	-0.66 [-1.35, 0.03]
Khadadah et al. 2022b	-0.5	0.81853528	17	-0.3	0.85440038	17	5.8%	-0.23 [-0.91, 0.44]
Klefbeck et al. 2003	-0.1	0.95283787	7	0.9	1.54415025	8	3.7%	-0.72 [-1.78, 0.34]
Mostert et al. 2002	-0.7	1.85202592	12	-0.2	1.7691806	13	5.1%	-0.27 [-1.06, 0.52]
Pfalzer et al. 2011	-1.4	10.28056419	20	-1.4	12.31868499	19	6.2%	0.00 [-0.63, 0.63]
**Subtotal (95% CI)**			**142**			**150**	**46.7%**	**-0.64 [-1.07, -0.20]**
Heterogeneity: Tau² = 0.29; Chi² = 24.52, df = 8 (P = 0.002); I² = 67%								
Test for overall effect: Z = 2.85 (P = 0.004)								
**Total (95% CI)**			**307**			**318**	**100.0%**	**-0.50 [-0.76, -0.24]**
Heterogeneity: Tau² = 0.18; Chi² = 41.94, df = 18 (P = 0.001); I² = 57%								
Test for overall effect: Z = 3.80 (P = 0.0001)								
Test for subgroup differences: Chi² = 0.68, df = 1 (P = 0.41), I² = 0%								

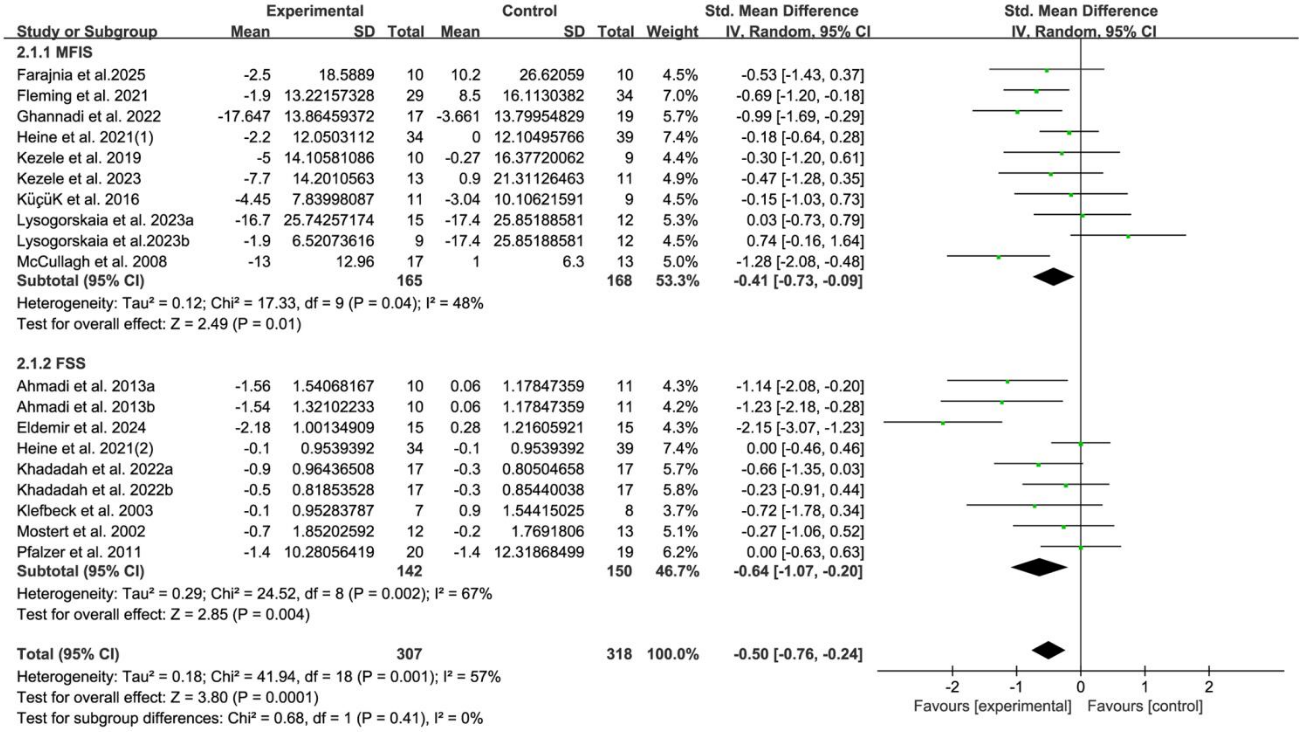

Supplementary Figure 5. Meta-analysis of the effect of type of fatigue test on fatigue in MS patients on fatigue in MS patients.

Study or Subgroup	Experimental Mean	Experimental SD	Experimental Total	Control Mean	Control SD	Control Total	Weight	Std. Mean Difference IV, Random, 95% CI
**4.1.1 aerobic training**								
Ahmadi et al. 2013a	-1.56	1.540682	10	0.06	1.178474	11	4.7%	-1.14 [-2.08, -0.20]
Farajnia et al.2025	-2.5	18.5889	10	10.2	26.62059	10	4.9%	-0.53 [-1.43, 0.37]
Heine et al. 2021(1)	-2.2	12.05031	34	0	12.10496	39	8.2%	-0.18 [-0.64, 0.28]
Heine et al. 2021(2)	-0.1	0.953939	34	-0.1	0.953939	39	8.2%	0.00 [-0.46, 0.46]
Kezele et al. 2019	-5	14.10581	10	-0.27	16.3772	9	4.9%	-0.30 [-1.20, 0.61]
Kezele et al. 2023	-7.7	14.20106	13	0.9	21.31126	11	5.4%	-0.47 [-1.28, 0.35]
Lysogorskaia et al.2023b	-1.9	6.520736	9	-17.4	25.85189	12	4.9%	0.74 [-0.16, 1.64]
McCullagh et al. 2008	-13	12.96	17	1	6.3	13	5.5%	-1.28 [-2.08, -0.48]
Mostert et al. 2002	-0.7	1.852026	12	-0.2	1.769181	13	5.6%	-0.27 [-1.06, 0.52]
**Subtotal (95% CI)**			**149**			**157**	**52.5%**	**-0.35 [-0.69, 0.00]**
Heterogeneity: Tau² = 0.14; Chi² = 16.28, df = 8 (P = 0.04); I² = 51%								
Test for overall effect: Z = 1.95 (P = 0.05)								
**4.1.2 Pilates**								
Eldemir et al. 2024	-2.18	1.001349	15	0.28	1.216059	15	4.8%	-2.15 [-3.07, -1.23]
Fleming et al. 2021	-1.9	13.22157	29	8.5	16.11304	34	7.8%	-0.69 [-1.20, -0.18]
KüçüK et al. 2016	-4.45	7.839981	11	-3.04	10.10622	9	5.0%	-0.15 [-1.03, 0.73]
**Subtotal (95% CI)**			**55**			**58**	**17.6%**	**-0.97 [-1.97, 0.03]**
Heterogeneity: Tau² = 0.63; Chi² = 10.38, df = 2 (P = 0.006); I² = 81%								
Test for overall effect: Z = 1.90 (P = 0.06)								
**4.1.3 Traditional breathing training**								
Ghannadi et al. 2022	-17.647	13.86459	17	-3.661	13.79955	19	6.3%	-0.99 [-1.69, -0.29]
Khadadah et al. 2022a	-0.9	0.964365	17	-0.3	0.805047	17	6.3%	-0.66 [-1.35, 0.03]
Khadadah et al. 2022b	-0.5	0.818535	17	-0.3	0.8544	17	6.5%	-0.23 [-0.91, 0.44]
Klefbeck et al. 2003	-0.1	0.952838	7	0.9	1.54415	8	4.1%	-0.72 [-1.78, 0.34]
Pfalzer et al. 2011	-1.4	10.28056	20	-1.4	12.31868	19	6.8%	0.00 [-0.63, 0.63]
**Subtotal (95% CI)**			**78**			**80**	**29.9%**	**-0.48 [-0.85, -0.11]**
Heterogeneity: Tau² = 0.04; Chi² = 5.25, df = 4 (P = 0.26); I² = 24%								
Test for overall effect: Z = 2.53 (P = 0.01)								
**Total (95% CI)**			**282**			**295**	**100.0%**	**-0.50 [-0.77, -0.23]**
Heterogeneity: Tau² = 0.18; Chi² = 37.83, df = 16 (P = 0.002); I² = 58%								
Test for overall effect: Z = 3.60 (P = 0.0003)								
Test for subgroup differences: Chi² = 1.40, df = 2 (P = 0.50), I² = 0%								

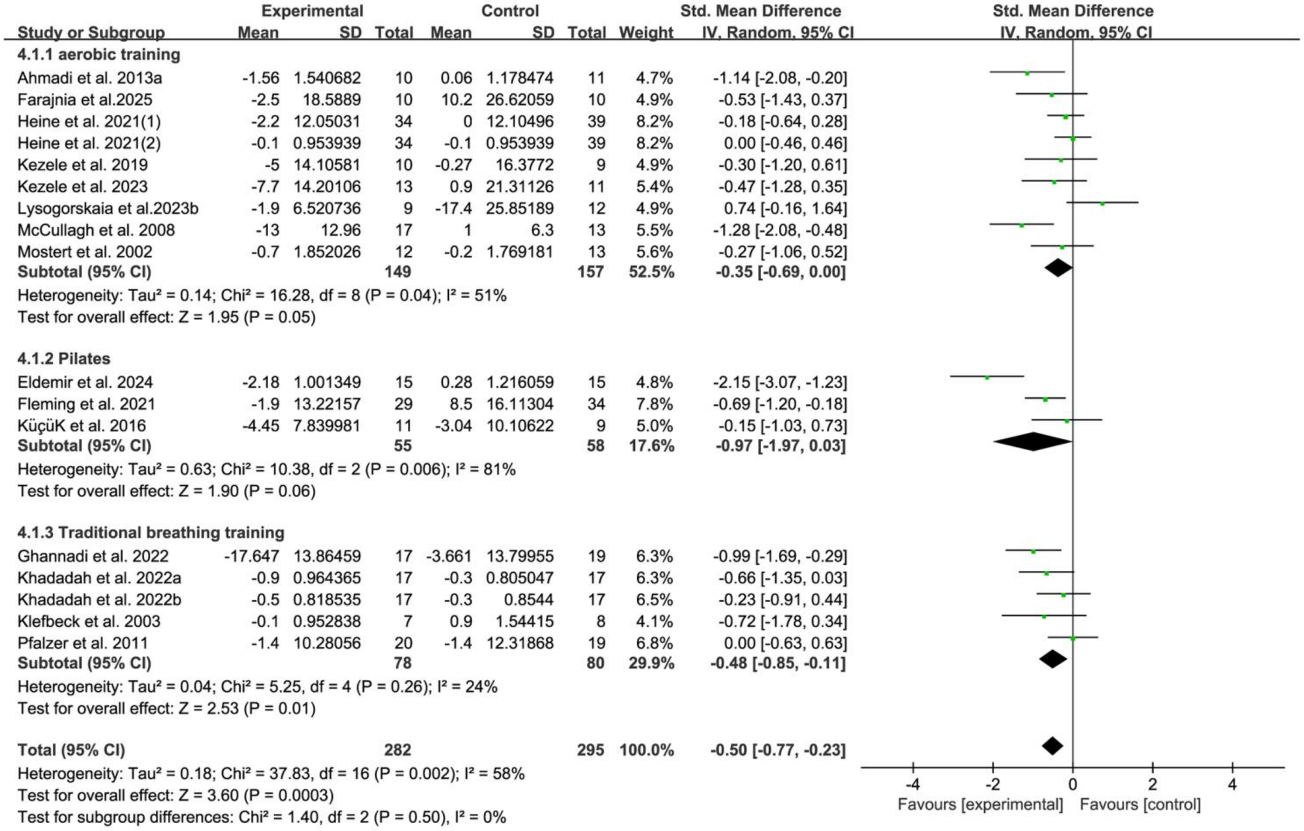

Supplementary Figure 6. Meta-analysis of the effect of type of intervention on fatigue in MS patients on fatigue in MS patients.

Study or Subgroup	Experimental Mean	Experimental SD	Experimental Total	Control Mean	Control SD	Control Total	Weight	Std. Mean Difference IV, Random, 95% CI
Ahmadi et al. 2013a	-1.56	1.540682	10	0.06	1.178474	11	4.0%	-1.14 [-2.08, -0.20]
Ahmadi et al. 2013b	-1.54	1.321022	10	0.06	1.178474	11	4.0%	-1.23 [-2.18, -0.28]
Eldemir et al. 2024	-2.18	1.001349	15	0.28	1.216059	15	0.0%	-2.15 [-3.07, -1.23]
Farajnia et al.2025	-2.5	18.5889	10	10.2	26.62059	10	4.3%	-0.53 [-1.43, 0.37]
Fleming et al. 2021	-1.9	13.22157	29	8.5	16.11304	34	8.2%	-0.69 [-1.20, -0.18]
Ghannadi et al. 2022	-17.647	13.86459	17	-3.661	13.79955	19	6.0%	-0.99 [-1.69, -0.29]
Heine et al. 2021(1)	-2.2	12.05031	34	0	12.10496	39	9.0%	-0.18 [-0.64, 0.28]
Heine et al. 2021(2)	-0.1	0.953939	34	-0.1	0.953939	39	9.0%	0.00 [-0.46, 0.46]
Kezele et al. 2019	-5	14.10581	10	-0.27	16.3772	9	4.2%	-0.30 [-1.20, 0.61]
Kezele et al. 2023	-7.7	14.20106	13	0.9	21.31126	11	4.9%	-0.47 [-1.28, 0.35]
Khadadah et al. 2022a	-0.9	0.964365	17	-0.3	0.805047	17	6.0%	-0.66 [-1.35, 0.03]
Khadadah et al. 2022b	-0.5	0.818535	17	-0.3	0.8544	17	6.2%	-0.23 [-0.91, 0.44]
Klefbeck et al. 2003	-0.1	0.952838	7	0.9	1.54415	8	3.4%	-0.72 [-1.78, 0.34]
KüçüK et al. 2016	-4.45	7.839981	11	-3.04	10.10622	9	4.4%	-0.15 [-1.03, 0.73]
Lysogorskaia et al. 2023a	-16.7	25.74257	15	-17.4	25.85189	12	5.4%	0.03 [-0.73, 0.79]
Lysogorskaia et al.2023b	-1.9	6.520736	9	-17.4	25.85189	12	4.3%	0.74 [-0.16, 1.64]
McCullagh et al. 2008	-13	12.96	17	1	6.3	13	5.0%	-1.28 [-2.08, -0.48]
Mostert et al. 2002	-0.7	1.852026	12	-0.2	1.769181	13	5.1%	-0.27 [-1.06, 0.52]
Pfalzer et al. 2011	-1.4	10.28056	20	-1.4	12.31868	19	6.7%	0.00 [-0.63, 0.63]
**Total (95% CI)**			**292**			**303**	**100.0%**	**-0.41 [-0.64, -0.19]**

Heterogeneity: Tau² = 0.09; Chi² = 28.35, df = 17 (P = 0.04); I² = 40%
Test for overall effect: Z = 3.67 (P = 0.0002)

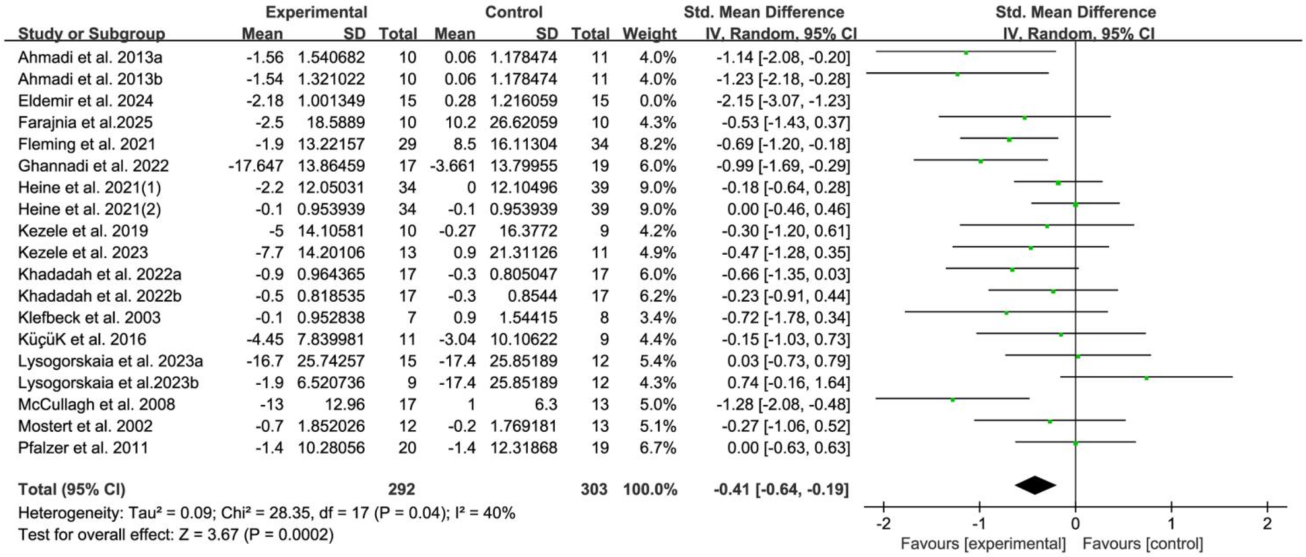

Supplementary Figure 7. Sensitivity analyses results of fatigue.

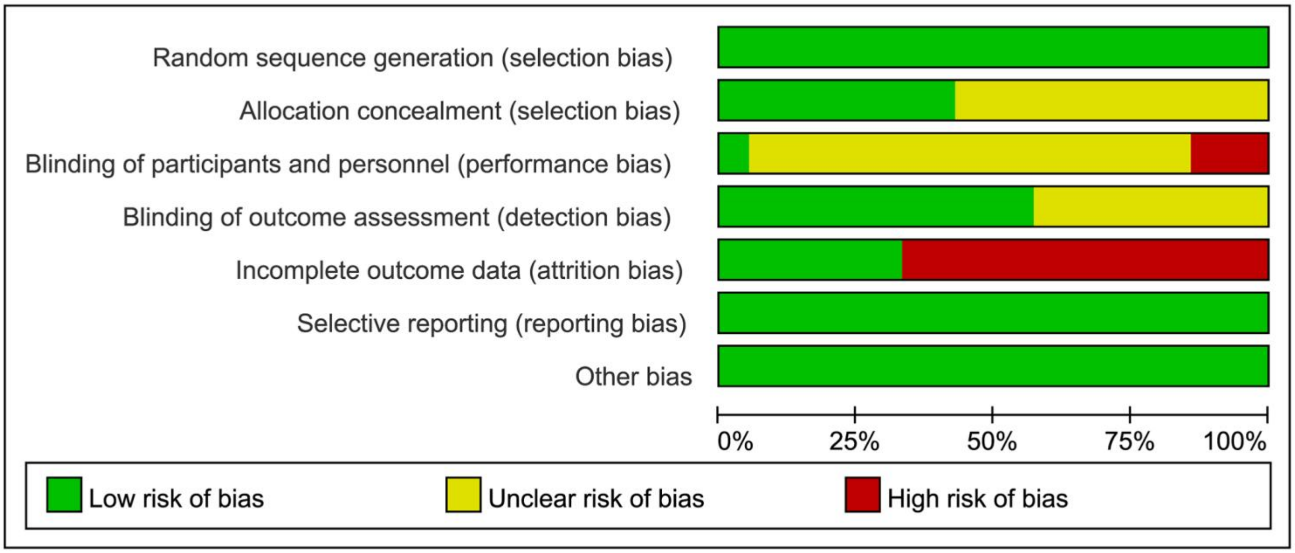

Supplementary Figure 8. Results of Cochrane risk of bias tool.

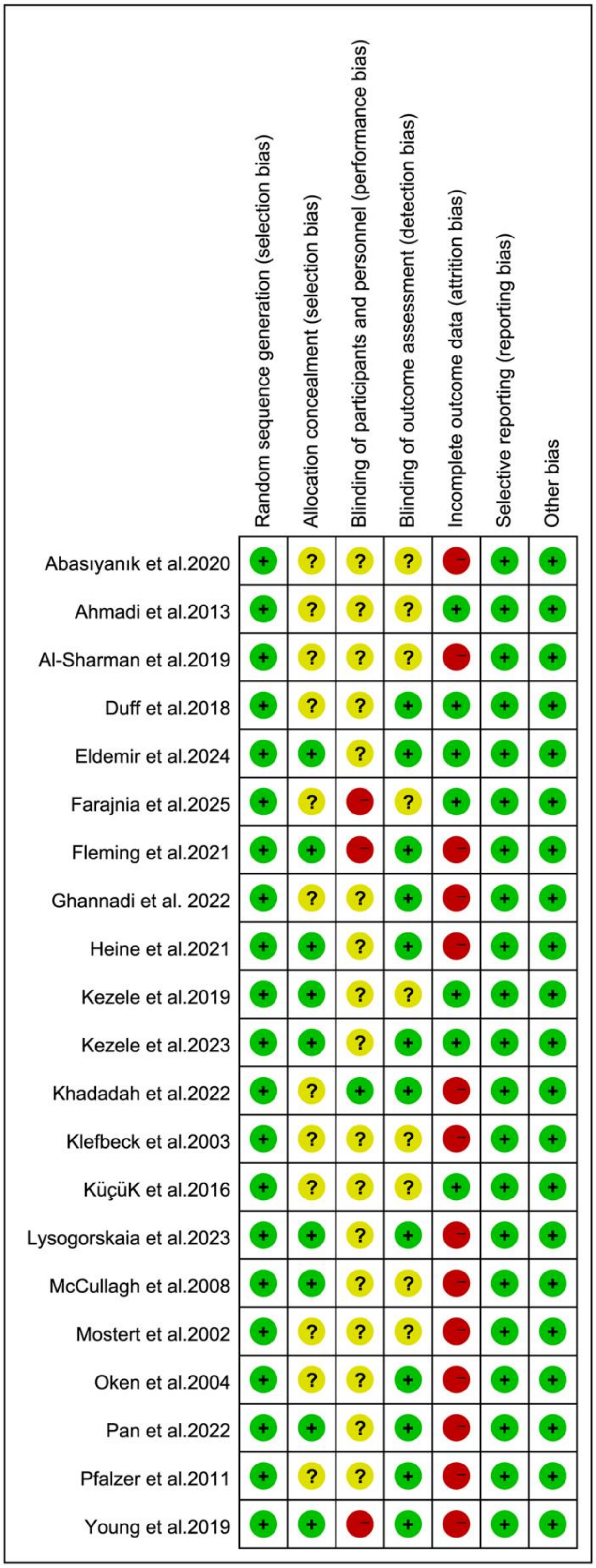

Supplementary Figure 9. Results of Cochrane risk of bias tool.

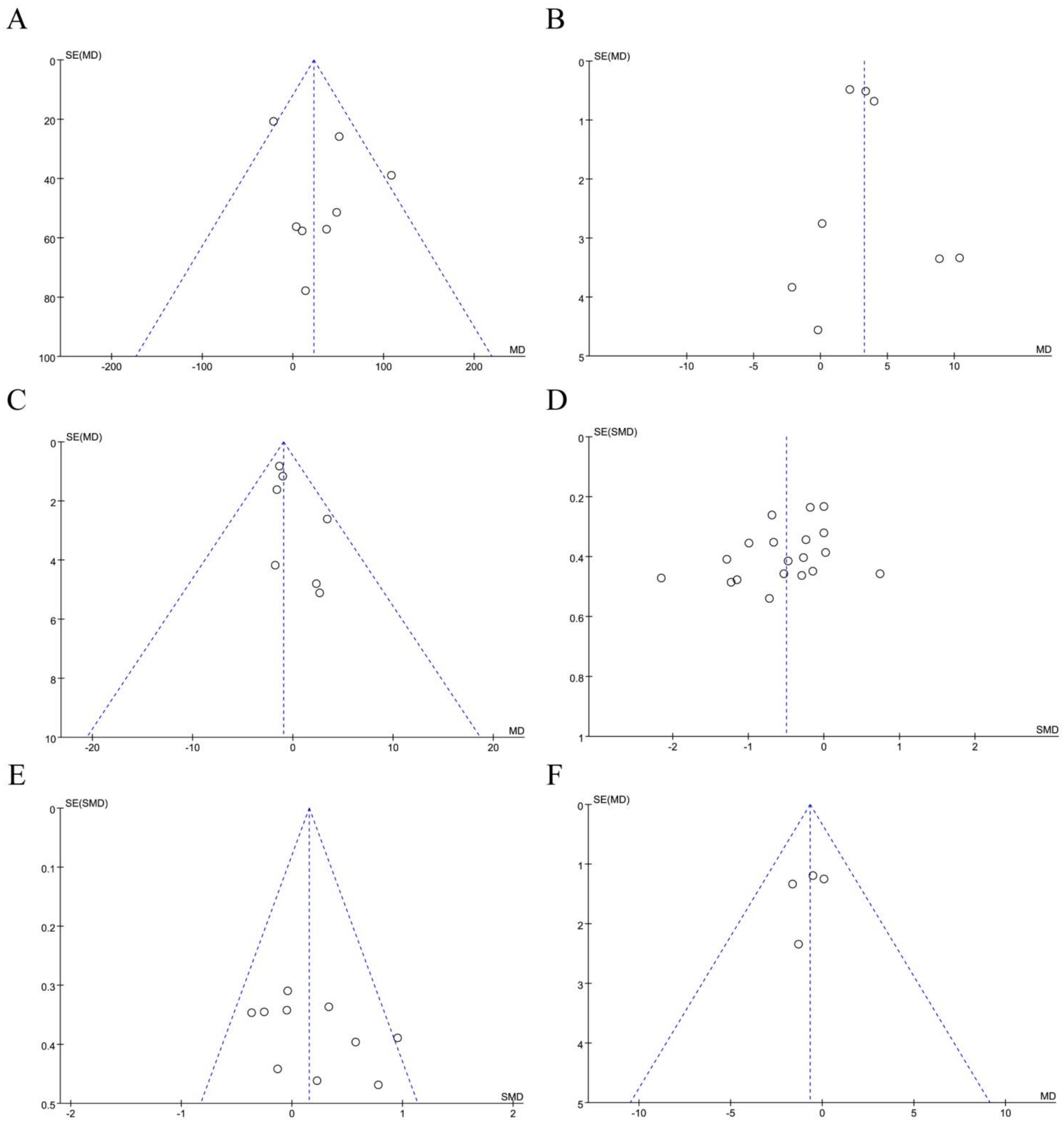

Supplementary Figure 10. Funnel plot. A. 6MWT(6-minute walk test); B. BBS(Berg Balance Scale); C. TUG(Timed Up and Go); D. Quality of life; E. Fatigue; F. PSQI(Pittsburgh sleep quality index).

2.2 Supplementary Tables

Study	A	B	C	D	E	F	G	H	I	J	K	Score
Lysogorskaia et al.2023	Y	1	1	1	0	0	1	0	1	1	1	7/10

Kezele et al.2023	Y	1	1	1	0	0	1	0	1	1	1	7/10
Oken et al.2004	Y	1	0	1	0	0	1	0	1	1	1	6/10
McCullagh et al.2008	Y	1	1	1	0	0	0	1	1	1	1	7/10
KüçüK et al.2016	Y	1	0	1	0	0	0	0	1	1	1	5/10
Duff et al.2018	Y	1	0	1	0	0	1	0	1	1	1	6/10
Fleming et al.2021	Y	1	1	1	0	0	1	0	1	1	1	7/10
Kezele et al.2019	Y	1	1	1	0	0	0	0	1	1	1	6/10
Eldemir et al.2024	Y	1	1	1	0	0	1	0	1	1	1	7/10
Young et al.2019	Y	1	1	1	0	0	1	0	1	1	1	7/10
Abasıyanık et al.2020	Y	1	0	1	0	0	0	0	1	1	1	5/10
Pan et al.2022	Y	1	1	1	0	0	1	0	1	1	1	7/10
Al-Sharman et al.2019	Y	1	0	1	0	0	0	0	1	1	1	5/10
Mostert et al.2002	Y	1	0	1	0	0	0	0	1	1	1	5/10
Farajnia et al.2025	Y	1	0	1	0	0	0	0	1	1	1	5/10
Pfalzer et al.2011	Y	1	0	1	0	0	1	0	1	1	1	6/10
Klefbeck et al.2003	Y	1	0	1	0	0	0	1	1	1	1	6/10
Ghannadi et al. 2022	Y	1	0	1	0	0	1	0	1	1	1	6/10
Khadadah et al.2022	Y	1	0	1	1	0	1	1	1	1	1	8/10
Heine et al.2021	Y	1	1	1	0	0	1	1	1	1	1	8/10
Ahmadi et al.2013	Y	1	0	1	0	0	0	0	1	1	1	5/10

Supplementary Table 1. Methodological assessment of randomized controlled trials included in the systematic review using the PEDro scale. A, eligibility criteria; B, random allocation; C, concealed allocation; D, baseline comparability; E, blind subjects; F, blind therapists; G, blind assessors; H, adequate follow-up; I, intention-to-treat analysis; J, between-group comparisons; K, point estimates and variability. The total score represents the score of the PEDro scale. Item 1 was not scored. Y: yes.
